# Molecular Analysis of Volatile Metabolites Synthesized by *Candida albicans* and *Staphylococcus aureus* in In Vitro Cultures and Bronchoalveolar Lavage Specimens Reflecting Single- or Duo-Factor Pneumonia

**DOI:** 10.3390/biom14070788

**Published:** 2024-07-02

**Authors:** Wojciech Filipiak, Matthias Wenzel, Clemens Ager, Chris A. Mayhew, Tomasz Bogiel, Robert Włodarski, Markus Nagl

**Affiliations:** 1 Department of Pharmacodynamics and Molecular Pharmacology, Faculty of Pharmacy, Collegium Medicum in Bydgoszcz, Nicolaus Copernicus University in Toruń, A. Jurasza 2 Str., 85-089 Bydgoszcz, Poland; 2 Institute for Breath Research, Universität Innsbruck, Innrain 66 and 80-82, A-6020 Innsbruck, Austria; matthias.wenzel.bellmann@gmail.com (M.W.); clemens.ager@uibk.ac.at (C.A.); christopher.mayhew@uibk.ac.at (C.A.M.); 3 Department of Microbiology, Faculty of Pharmacy, Collegium Medicum in Bydgoszcz, Nicolaus Copernicus University in Toruń, Maria Curie-Skłodowska 9 Str., 85-094 Bydgoszcz, Poland; t.bogiel@cm.umk.pl; 4 Department of Anaesthesiology and Intensive Care, 10th Military Research Hospital and Polyclinic, Powstańców Warszawy 5 Str., 85-681 Bydgoszcz, Poland; robert.wlodarski@10wsk.mil.pl; 5 Institute of Hygiene and Medical Microbiology, Medical University of Innsbruck, Schöpfstr. 41, A-6020 Innsbruck, Austria; m.nagl@i-med.ac.at

**Keywords:** bacterial metabolites, bronchoalveolar lavage sampling, cross-kingdom interactions, thin-film microextraction, ventilation-associated pneumonia diagnosis, volatile biomarkers

## Abstract

Current microbiological methods for pneumonia diagnosis require invasive specimen collection and time-consuming analytical procedures. There is a need for less invasive and faster methods to detect lower respiratory tract infections. The analysis of volatile metabolites excreted by pathogenic microorganisms provides the basis for developing such a method. Given the synergistic role of *Candida albicans* in increasing the virulence of pathogenic bacteria causing pneumonia and the cross-kingdom metabolic interactions between microorganisms, we compare the emission of volatiles from *Candida albicans* yeasts and the bacteria *Staphylococcus aureus* using single and mixed co-cultures and apply that knowledge to human in vivo investigations. Gas chromatography–mass spectrometry (GC-MS) analysis resulted in the identification of sixty-eight volatiles that were found to have significantly different levels in cultures compared to reference medium samples. Certain volatiles were found in co-cultures that mainly originated from *C. albicans* metabolism (e.g., isobutyl acetate), whereas other volatiles primarily came from *S. aureus* (e.g., ethyl 2-methylbutyrate). Isopentyl valerate reflects synergic interactions of both microbes, as its level in co-cultures was found to be approximately three times higher than the sum of its amounts in monocultures. Hydrophilic–lipophilic-balanced (HLB) coated meshes for thin-film microextraction (TFME) were used to preconcentrate volatiles directly from bronchoalveolar lavage (BAL) specimens collected from patients suffering from ventilation-associated pneumonia (VAP), which was caused explicitly by *C. albicans* and *S. aureus*. GC-MS analyses confirmed the existence of in vitro-elucidated microbial VOCs in human specimens. Significant differences in BAL-extracted amounts respective to the pathogen-causing pneumonia were found. The model in vitro experiments provided evidence that cross-kingdom interactions between pathogenic microorganisms affect the synthesis of volatile compounds. The TFME meshes coated with HLB particles proved to be suitable for extracting VOCs from human material, enabling the translation of in vitro experiments on the microbial volatilome to the in vivo situation involving infected patients. This indicates the direction that should be taken for further clinical studies on VAP diagnosis based on volatile analysis.

## 1. Introduction

According to the National Healthcare Safety Network, a tracking system from the Centers for Disease Control and Prevention, more than 15 thousand pathogens causing ventilator-associated pneumonia (VAP) were reported from 1155 Acute Care Hospitals in the USA up to 2021, with *Staphylococcus aureus* being the most prevalent pathogen (29.6%), followed by *Pseudomonas aeruginosa* (13.4%) [1]. Notably, both pathogens interact with *Candida* spp., a yeast that readily colonizes the airways in more than 50% of patients with suspected VAP [2]. These fungal–bacterial interactions promote the growth of antibiotic-resistant bacteria [2,3,4,5] and enhance their virulence [6,7,8]. Hence, VAP patients colonized with *Candida* spp. are at risk of prolonged mechanical ventilation, extended ICU stay, and increased mortality [2,3,9,10,11]. As pointed out by Ricard and Roux, taking together all experimental and clinical clues, *Candida* spp. should no longer be considered a bystander [12]. Instead, it may constitute leads for new therapies and early non-invasive diagnosis of pneumonia, triggering an early workup and potentially providing methods for monitoring the response to treatment. Such a promising diagnostic technique utilizes volatile organic compounds (VOCs) produced by microorganisms that can be identified and quantified in the exhaled breath gas of patients using mass spectrometry, either offline after sample preconcentration and chromatographic analysis in the laboratory [13,14,15] or in real time directly from mechanically ventilated patients [16]. Analysis of bacterial VOCs in exhaled breath has excellent applicability in VAP because it is entirely non-invasive and can be performed repeatedly without any burden to the patient. Hence, the analysis of volatiles in exhaled breath could serve as a non-invasive and sensitive supportive method to the established invasive (tracheal aspirates or bronchoalveolar lavage sampling) and a time-intensive procedure of microbiological testing. Ultimately, once the key volatiles have been correctly identified, an analytical analysis could be performed using portable point-of-care devices, such as sensors, leading to timely, effective, and personalized antibiotic use, so much needed to deescalate the problem of antimicrobial resistance [17,18].

So far, both discussed species have been investigated in terms of VOC release multiple times but as individual cultures grown under in vitro conditions. The reader can find a comprehensive review summarizing these findings elsewhere [17]. There is a scarce number of studies directly comparing VOC production by both *C. albicans* and *S. aureus*. In this regard, Karami et al. [19] demonstrated that apart from compounds produced by both microorganisms cultured in vitro, some species-specific metabolites could also be detected, with alcohols being the most frequently observed for *C. albicans,* and there was a larger diversity for *S. aureus*. In turn, Rees and colleagues [20] used two-dimensional gas chromatography time-of-flight mass spectrometry to analyze VOCs in the headspace of 100 clinical isolates covering bloodstream or urinary tract infections (including eight samples of *C. albicans* and ten samples of *S. aureus*) and successfully discriminating these pathogens based on a VOC selected with machine learning algorithms. While very interesting studies that focused on inter-species interactions between *S. aureus* and *C. albicans* cultured together are available, demonstrating their importance to the increased virulence [21] and drug resistance [22] of both discussed pathogens, nothing is known about their impact on the secretion of volatile organic compounds.

Therefore, the aim of the first part of this study is to identify the volatile metabolites excreted by *C. albicans* and *S. aureus* in single cultures and mixed co-cultures. Mixed co-cultures are investigated to determine whether the cross-kingdom interactions between these two species manifest in specific VOC profiles. For this purpose, separate bacterial, fungal, and mixed cultures were established under strictly controlled conditions of temperature, humidity, and ventilation. Dynamic headspace sampling on multibed sorption tubes was performed at several time points to follow the changes in VOC profiles. The aim of the second part of our work is to reveal whether volatiles found under in vitro conditions (model study) are also present in vivo (clinical samples). Therefore, bronchoalveolar lavage (BAL) specimens were collected from mechanically ventilated patients coinfected explicitly with the two mentioned pathogens. Due to the small quantity of the biological material (BAL), statical headspace (HS) sampling needs to be applied to preconcentrate VOCs before gas chromatographic–mass spectrometric (GC-MS) analysis. For this purpose, novel thin-film microextraction (TFME) devices with a mesh-like geometry are used which have a larger stationary phase area-to-volume ratio compared to the regular solid-phase microextraction (SPME) fibers. The resulting enhanced extraction efficiency is further improved for oxygenated VOCs (aldehydes, ketones, alcohols) by the application of the hydrophilic–lipophilic-balanced (HLB) polymer particles, enabling the trace analysis (down to the sub-ppb level) of the polar volatile metabolites [23], which are also important also for microorganisms.

## 2. Materials and Methods

### 2.1. Chemicals

Liquid and gaseous chemicals manufactured by Acros Organics, Alfa Aesar, Honeywell (which all belong to the Thermo Scientific Chemicals, Waltham, MA, USA), Sigma-Aldrich (Merck KGaA, Darmstadt, Germany) or Tokyo Chemical Industry (Tokyo, Japan) were purchased from AlChem (Toruń, Poland). Broth Medium (Caso Bouillon from Merck, Darmstadt, Germany) was prepared as recommended by Merck, and 30 g of medium powder was dissolved in an Erlenmeyer flask with distilled water up to 1 L. The broth medium consists of casein peptone 17.0 g/L, peptone from soya flour 3.0 g/L, D + glucose 2.5 g/L, sodium chloride 5.0 g/L, and di-potassium hydrogen phosphate 2.5 g/L. The culture medium was decanted into four 100 mL cleaned culture flasks.

### 2.2. Pre-Culture Preparation and Colony-Forming Unit Counting

All in vitro experiments with fungal and bacterial cultures were performed at the Institute of Hygiene and Medical Microbiology, Medical University of Innsbruck. The collected samples were then analyzed at the Institute for Breath Research, Universität Innsbruck.

Stock cultures of *Candida albicans* (CBS 5982) and *Staphylococcus aureus* (ATCC 6538) were stored at 4 °C on Müller–Hinton agar plates (CM0337 Oxoid Ltd., Hampshire, Great Britain). Single colonies from these were inoculated into 5 mL of the medium (Caso Bouillon, Merck Darmstadt) and grown overnight at 37 °C (Memmert oven, Loading Modell 100–800, 91,107 Schwabach Germany) to approximately 1 × 10^7^ colony-forming units (CFUs)/mL (*C. albicans*) and 3 × 10^9^ CFUs/mL (*S. aureus*). To investigate VOC production in a single culture and co-culture, aliquots from the overnight culture of each microbe were inoculated in appropriate quantities into 100 mL of the medium to yield the required concentration (approximately 1 × 10^5^ CFUs/mL for *C. albicans* and 1 × 10^3^ CFUs/mL for *S. aureus*). A magnetic stirrer circulating the liquid culture at 50 rpm enabled the oxygen uptake and VOC release into the medium headspace.

At each considered time point, 1 mL of medium suspension was collected to count microorganisms. Aliquots (50 µL per plate) of undiluted samples and samples diluted with 0.9% NaCl (10^−2^–10^−6^ -fold) were plated on Müller–Hinton agar plates with an automated spiral plater (model WASP2, Don Whitley Scientific, Shipley, London, UK). CFU counts were performed after incubation over 24 h at 37 °C. Morphological differences, primarily the color of *C. albicans* (white) and *S. aureus* (yellowish), allowed both species of co-cultures to be distinguished.

### 2.3. Experimental Setup for Cultivating and Headspace Sampling

An in-house system that has been previously described in detail was used [24]. Briefly, four glass bottles containing 100 mL of liquid sample each (either microbe or sterile reference medium) were kept within a water bath at 37 °C and gently stirred (50 rpm). The incubation setup was the following. Flask No. 1 was the control medium (sterile, without microorganisms), flask No. 2 was a sole *C. albicans* culture, flask No. 3 was a sole *S. aureus* culture, and flask No. 4 contained co-cultured *C. albicans* and *S. aureus*. To sample the headspace above a liquid, synthetic air flowed over the medium. This air had a purity of 5.5, was enriched with 5% CO_2_ (Linde, Stadl-Pradl, Austria), and additionally, was purified with a Supelcarb™ hydrocarbon trap (Supelco, Bellefonte, PA, USA) and an inline catalyst (Parker Zero Air Generator, Balston^®^, model: 75-83-220, Parker Hannifin Corporation, Haverhill, MA, USA). To decrease the high relative humidity that could lead to undesired condensation in transfer lines or excessive water uptake on the adsorption tube, the entire system was placed in the incubator at 45 °C, and an additional flow of purified synthetic air was used (35 mL/min) to dilute the headspace sample (5 mL/min). All the flows in four cultures were precisely controlled and additionally checked at the outlet of a sorption tube using a flowmeter (Red-Y Compact GCR, Vögtlin Instruments AG, Aesch; Switzerland). For single sampling, 200 mL of the gas flow was adsorbed on a tube filled with Carbotrap B and Carbopack X within a 40-minute timeframe. The first gas samples were collected before the inoculation of the microorganisms to assess the volatile compounds present in the headspace of a sterile medium after purging overnight with synthetic air (which considerably reduces background VOCs, as demonstrated in a previous study [25]). Time points at 2 h, 3.5 h, 5 h, 6.5 h, 8 h, 26 h, and 28 h were chosen for headspace sampling based on microorganisms’ growth rate. One mL of the culture suspension for CFU counting was collected immediately after the headspace sampling using a slightly bent 12 cm needle (Sterican) introduced via a septum port (ensuring that the system remains gas tight). The entire experiment—starting from the collection of headspace gas from the medium still before inoculation of microorganisms until the last time point—was replicated six times.

### 2.4. Collection and Preparation of Bronchoalveolar Lavage Specimens

All BAL samples were collected from mechanically ventilated patients hospitalized in the Anesthesiology and Intensive Care Unit of the 10th Military Research Hospital and Polyclinic in Bydgoszcz, Poland. Subsequent TFME-GC-MS analyses of BAL specimens were performed at the Department of Pharmacodynamics and Molecular Pharmacology, Nicolaus Copernicus University in Toruń, Poland. Ethical approval for this study was obtained from the local Bioethics Committee (KB-218/2018). The patients enrolled in this study had to fulfill the following inclusion criteria: (1) admission to the ICU, (2) mechanically ventilated, and (3) suspected of or confirmed for VAP (based on clinical and microbiological evidence). The exclusion criteria were (1) age under 18, (2) pregnancy, (3) confirmed co-existing lung disease (traumatic lung injury or pulmonary cancer), (4) strict isolation at the ICU, (5) increased intracranial pressure (ICP), (6) positive end-expiratory pressure (PEEP) > 10, and (7) extra corporal heart and lung assistance devices.

According to the routine clinical practice in the Anesthesiology and Intensive Care Unit of the 10th Military Research Hospital and Polyclinic in Bydgoszcz (Poland), BAL samples were taken from mechanically ventilated patients for microbiological testing upon suspicion of pneumonia based on clinical evidence and tailoring the antimicrobial therapy. In accordance with the applied standard microbiological procedure, BAL inoculation was performed with a calibrated loop with a volume of 10 microliters on a set of media plates. The purulent part of the material was selected for culture. The residual BAL specimen was immediately transported from the Hospital Microbiology Laboratory to the University Analytical Laboratory for GC-MS analysis (both in Bydgoszcz, Poland). Meanwhile, the plated cultures were kept overnight at 37 °C with the following quantitative read out where a growth of at least 10^4^ CFUs/mL was considered diagnostic of the pathogen, which was further identified and subjected to antimicrobial susceptibility testing.

Altogether, as many as 92 BAL specimens were analyzed with TFME-GC-MS. However, for consistency with the in vitro experiments, this study only included those BAL samples for which microbiological testing confirmed the presence of either sole *C. albicans*, sole *S. aureus,* or both microbes and a lack of any others (resulting in the number of BAL samples of n = 6, n = 3, and n = 4, respectively, collected from 13 different patients).

### 2.5. Headspace Sampling from Bronchoalveolar Lavage Specimens

TFME meshes covered with the hydrophilic–lipophilic-balanced (HLB) stationary phase were manufactured according to the protocol developed by Grandy et al. [26] and were kindly provided by Prof Pawliszyn (University of Waterloo, Waterloo, ON, Canada). Before each sampling, the membranes were placed in an empty glass sorption tube (Supelco, Merck, Darmstadt, Germany) and preconditioned by putting them into a TD Clean-Cube conditioning unit (Scientific Instruments Manufacturer GmbH, Oberhausen, Germany). They were heated to a temperature of 250 °C for 30 min under a continuous flow of nitrogen 6.0 that had been further purified on a Carrier Gas Purifier (Agilent, Santa Clara, CA, USA).

Immediately after sample transport from the Hospital Microbiological Laboratory, 5 mL of each BAL specimen was placed into a 20 mL glass vial and warmed within an incubator up to 37 °C for 30 min. The preconcentration of VOCs secreted from the BAL sample was performed according to the previously optimized protocol [23]. Briefly, TFME sheets were suspended on a metal clamp under the cap with silicon septum inside the 20 mL glass vial containing the sample. Extraction took place at 37 °C for 90 min. Afterward, the TFME mesh was removed from the vial and placed into an empty sorption glass tube tightly closed in a TDS 3TM Storage Container (Supelco, Merck, Darmstadt, Germany) until the GC-MS analysis (performed on the day of headspace sampling).

### 2.6. Gas Chromatographic–Mass Spectrometric Analysis

For the in vitro experiments with microorganisms, the TD-GC-MS analyses were performed on a 6890N gas chromatograph with detection by the mass selective detector 5973N (both from Agilent Technologies, Waldbronn, Germany). Samples that were loaded on multibed sorption tubes were thermally desorbed at 300 °C over 10 min in a TDS3 unit equipped with a TDSA2 autosampler (both from Gerstel, Mulheim an der Ruhr, Germany). Cryofocusing during primary desorption was achieved at −90 °C in the CIS4 injector containing a trap filled with Carbotrap B (Gerstel, Mulheim an der Ruhr, Germany). The secondary desorption (i.e., injection into the GC column) was performed in splitless mode at 320 °C over 2 min. To ensure the best possible separation of sample constituents, the Rt-Q-Bond capillary column 30 m × 0.32 mm × 10 µm (Restek, Bellefonte, PA, USA) was used with the following temperature program: initial 55 °C held for 6 min, which was then ramped 7 °C/min to 97 °C (2 min), 2 °C/min to 110 °C (0 min), 5 °C/min to 130 °C (4 min), 5 °C/min to 160 °C (4 min), 4 °C/min to 230 °C (0 min), and 10 °C/min to 280 °C (4 min). For MS acquisition, the ionization energy was set to 70 eV, the ion source and quadrupole temperatures were 230 °C and 105 °C, respectively, and scanning was performed over a range of 20–200 *m*/*z*. The identification of detected analytes was carried out in two stages. A preliminary identification was obtained by matching the recorded mass spectrum to the NIST library. The identification was further confirmed through chromatographic parameters (retention time) of the respective standards. If particular standards were not available, the compound is accordingly marked. For quantitative analysis, a specialized software, BreathViewer 2.1, was used (ONCOTYROL, Center for Personalized Cancer Medicine GmbH, Innsbruck, Austria). This post-processing program enables the evaluation of the complex GC-MS data utilizing signal deconvolution and baseline correction to identify and quantify detected analytes. The final statistical calculations and plotting were performed with GraphPad Prism5 software.

For the analysis of BAL specimens, the TFME meshes with extracted analytes were thermally desorbed at 250 °C over 10 min in a TD-30R autosampler (Shimadzu, Shim-Pol, Warsaw, Poland). The cryofocusing during preliminary desorption was achieved at −20 °C on a cold trap filled with Carboxen. Rapid heating of the cold trap to 350 °C triggered injection into a Nexis 2030 Gas Chromatograph (Shimadzu, Shim-Pol, Warsaw, Poland) in splitless mode over 2 min. Sample constituents were separated on an Rt-Q-Bond capillary column 30 m × 0.25 mm × 8 µm (Restek, Bellefonte, PA, USA) using the oven temperature program developed for in vitro experiments (with only a slight modification of a final ramp). Data were acquired with a QP-2020-NX Mass Spectrometer (Shimadzu, Shim-Pol, Warsaw, Poland) operating in a SCAN mode within a range of 33–235 *m*/*z*. Analytes detected were identified using the same two-stage approach (NIST spectra library match and confirmation with standards) as mentioned above. Again, if a particular standard was not available, the compound was accordingly marked. For data processing, Shimadzu GCMS PostRun Analysis software was used for automatic peak detection and integration based on the previously established database of analytes and was further manually corrected (if necessary) by an experienced GC-MS analyst. To avoid errors in data interpretation caused by background contributions, the “Target Ion” was assigned to each analyte to integrate correctly the area under the chromatographic peak. Those “Target Ions” were selected manually by the experienced analyst to ensure either the most selective ion (unique for only one compound in the case of coeluted peaks) or the most sensitive ion (for peaks resolved to the baseline). Further, characteristic reference ions were assigned to each analyte to verify the peak identity during its integration process. The final statistical calculations and plotting were performed with Metaboanalyst 5.0 online software.

## 3. Results

### 3.1. Microbial Growth

**Figure 1:**
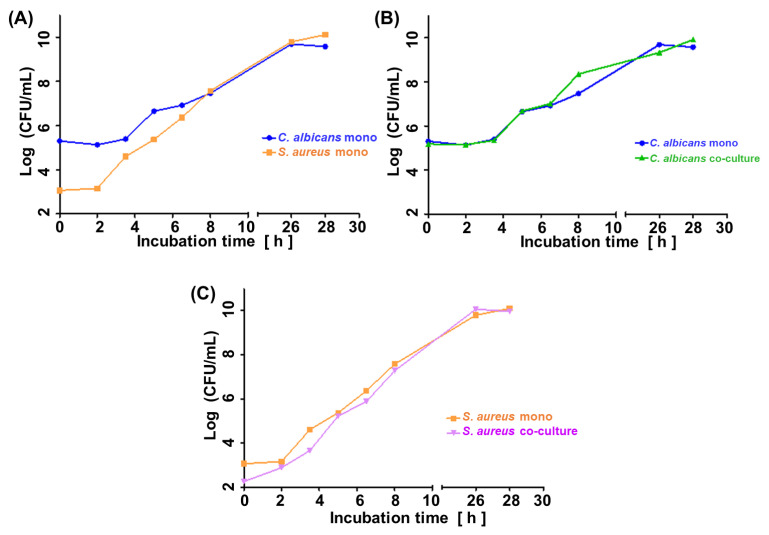
Growth curves of (**A**) single cultures of *C. albicans* and *S. aureus*, (**B**) *C. albicans* in a single and co-culture, (**C**) *S. aureus* in a single and co-culture. C.a.—*Candida albicans*; S.a.—*Staphylococcus aureus*.

Figure 1A illustrates the growth curve of *C. albicans* and *S. aureus* in monocultures. Given the shorter lag phase and faster proliferation rate of the second, its initial inoculum was adjusted two orders of magnitude lower (ca. 1 × 10^3^ CFUs/mL for *S. aureus* vs. ca. 1 × 10^5^ CFUs/mL for *C. albicans*). The shift in microbes’ initial quantities resulted in similar amounts of both species in the late log phase (8 h) and steady phase (26 h, 28 h) (Figure 1A), enabling a direct comparison of observed VOC profiles. Figure 1B,C demonstrate convergence in the growth rate of respective species in their single and co-cultures. There was no difference in the course of CFUs/mL from the start over the log to the stationary phase between the single and co-cultures of both species.

### 3.2. VOCs of Microbial Origin

By comparing the peak areas for respective analytes measured at the same time points in the headspace of cultures and sterile reference medium, several volatiles were found at significantly decreased levels in cultures, implicating their consumption by the microbes. Other VOCs appeared unaffected by microbial cultures, and their profile did not change throughout the experiment. However, a third group of compounds present at an initially low level either showed an increased concentration (temporarily or continuously) or, after the initial absence in the culture, they appeared during microbial growth. All VOCs with a significantly increased or decreased concentration in the culture headspace compared to the control medium headspace are given in Table 1, along with their parameters used for chromatographic identification (i.e., retention time, target ion, reference ions) and microbial synthesis or uptake status.

Since the VOC production in all cultures investigated in this study changed dynamically, they could be further split according to the microbial growth phase, distinguishing VOCs significantly higher in the log phase (3.5–8 h of cultivation) or the steady phase (24–26 h of cultivation). In this respect, *C. albicans* produced 28 VOCs and consumed 10 VOCs during the log phase (Supplementary Figure S1), while 20 volatiles were released and 14 were taken up during the steady phase (Supplementary Figure S2). In contrast, the metabolic activity of *S. aureus* was noticeably lower during the log phase, resulting in only five VOCs released and one VOC taken up (Supplementary Figure S3), but this increased proportionally to the bacterial load, yielding twenty-six VOCs produced and eleven VOCs consumed during the steady phase (Supplementary Figure S4). The highest metabolic activity was observed for the co-cultures of both species, where as many as 29 compounds were synthesized and 12 compounds were catabolized during the log phase (Supplementary Figure S5). Similarly, 25 metabolites were produced, and 19 were degraded in the steady phase of co-culture growth (Supplementary Figure S6).

### 3.3. Co-Culture-Specific VOCs

**Figure 2:**
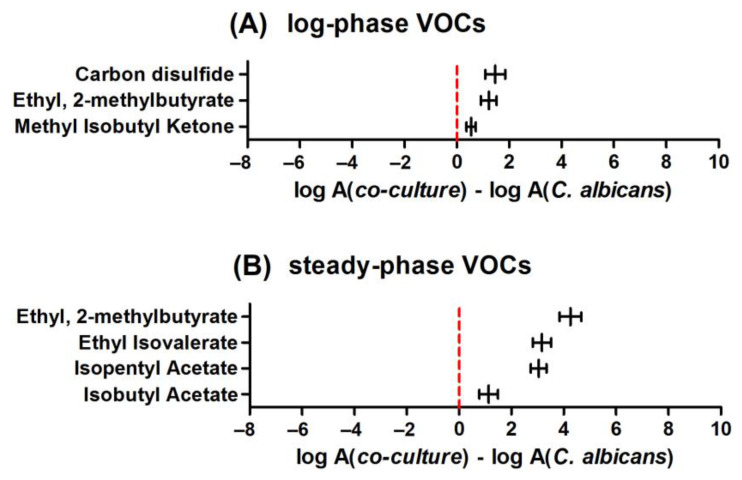
Relative abundance of VOCs ± standard error (horizontal whiskers) in the co-culture compared to the monoculture of *Candida albicans* given separately for the log phase (**A**) and the steady phase (**B**) of microbial growth. Positive values indicate a higher amount of respective VOCs in the co-culture than in the single culture.

To reveal the metabolic changes caused by inter-species’ interactions, the time-dependent profiles of VOC synthesis in co-cultures were compared to monocultures. For this purpose, VOCs with significantly different abundances in co-cultures and monocultures were normalized through log transformation and further subtracted. The positive value of such subtraction indicates a higher abundance of particular analytes in the co-culture, while a negative value indicates a lower abundance in the co-culture (and higher in a single culture). Similar to the previous considerations, these calculations were also performed separately for the log and steady phases of microbial growth. Figure 2 demonstrates differences in VOC profiles between *C. albicans* cultured alone and cultured with *S. aureus*. In the log phase, no single VOC could be found at a significantly higher level in the monoculture, whereas three volatiles showed a significantly higher production rate in the co-culture. An analogous comparison in the stationary phase confirmed the presence of four increasing and no significantly decreasing volatiles in the co-culture headspace.

**Figure 3:**
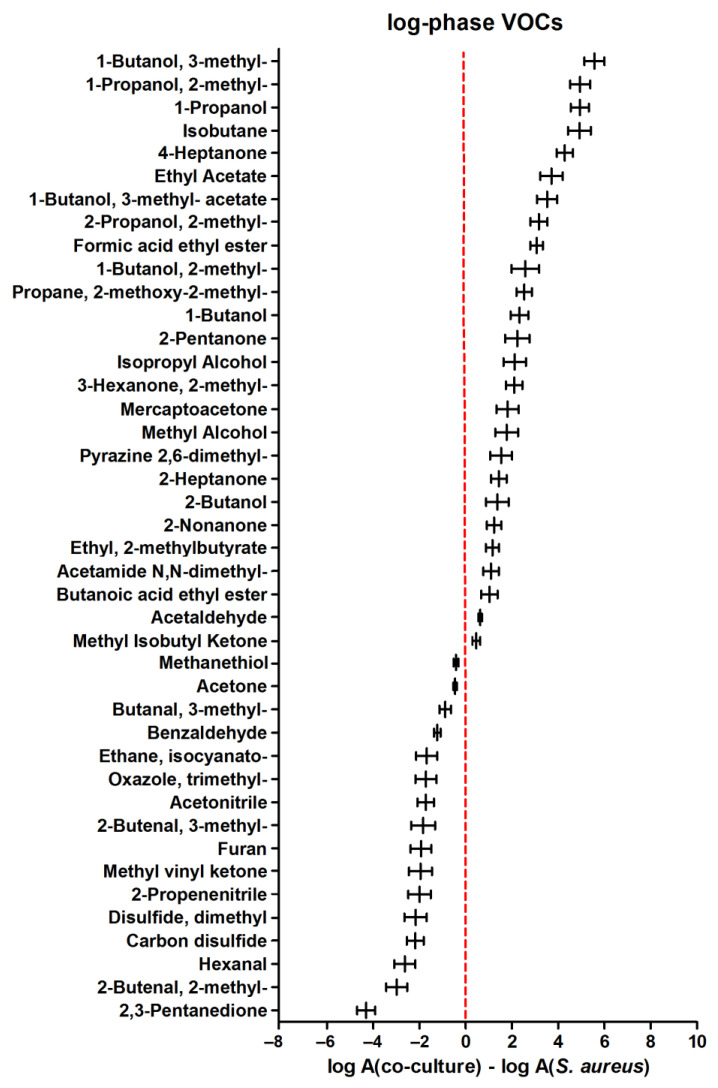
Relative abundance of VOCs ± standard error (horizontal whiskers) in a co-culture compared to a monoculture of *Staphylococcus aureus* in the log phase of growth. Positive values indicate a higher amount of respective VOCs in a co-culture than in a single culture of *S. aureus*, and vice versa; negative values indicate that metabolites are more abundant in a monoculture compared to a co-culture.

A comparison of VOC metabolism in the co-culture to the sole *S. aureus* during the log phase of microbial growth revealed 26 compounds significantly more abundant in the mixed culture headspace (those with positive values in Figure 3). Another 16 compounds were synthesized stronger in the *S. aureus* monoculture during the log phase.

**Figure 4:**
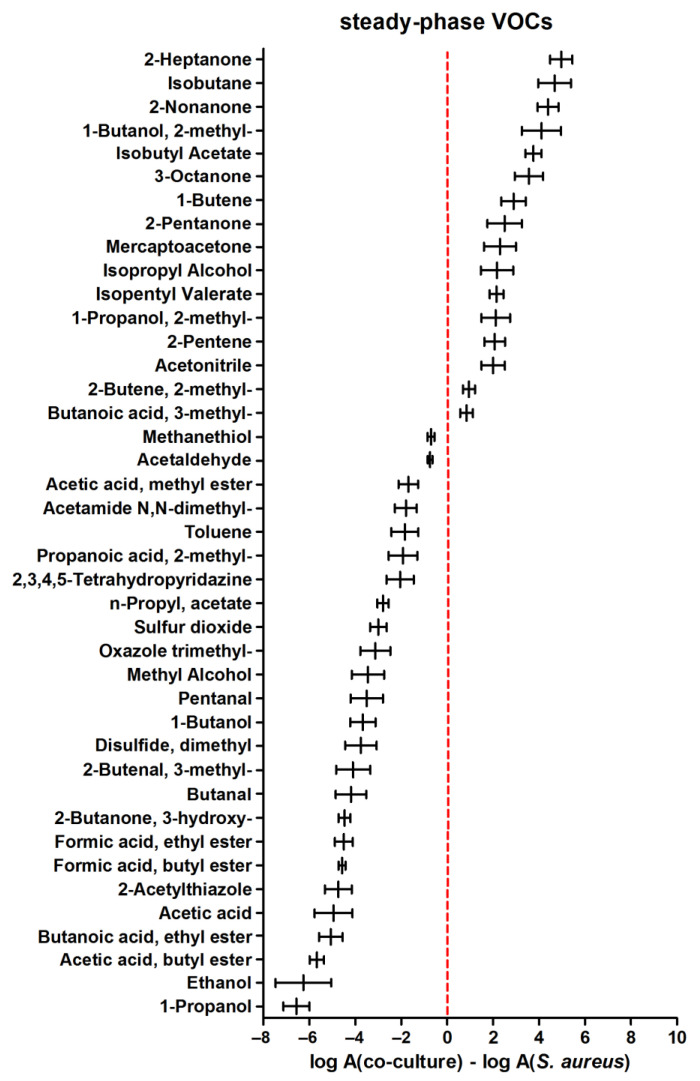
Relative abundance of VOCs ± standard error (horizontal whiskers) in a co-culture compared to a monoculture of *Staphylococcus aureus* in the steady phase of growth. Positive values indicate a higher amount of respective VOCs in a co-culture than in a single culture, and vice versa; negative values indicate that metabolites are more abundant in a monoculture compared to a co-culture.

In the later phase of microbial growth, higher metabolic activity of the sole *S. aureus* was more pronounced since as many as 25 volatiles were found at higher levels during the steady phase of *S. aureus* compared to its co-culture with *C. albicans* (Figure 4). At this stage, only 16 metabolites were synthesized in higher quantities in co-cultures (those with positive values in Figure 4).

For a selection of volatile metabolites that could testify about the interactions between co-existing *C. albicans* and *S. aureus*, the following inclusion criteria were chosen: (i) a significant change in the abundance of a particular compound relative to medium either in a log phase or in a steady phase and (ii) a significant change in the abundance of this analyte in the headspace of a co-culture relative to a single culture at the same stage of growth.

**Figure 5:**
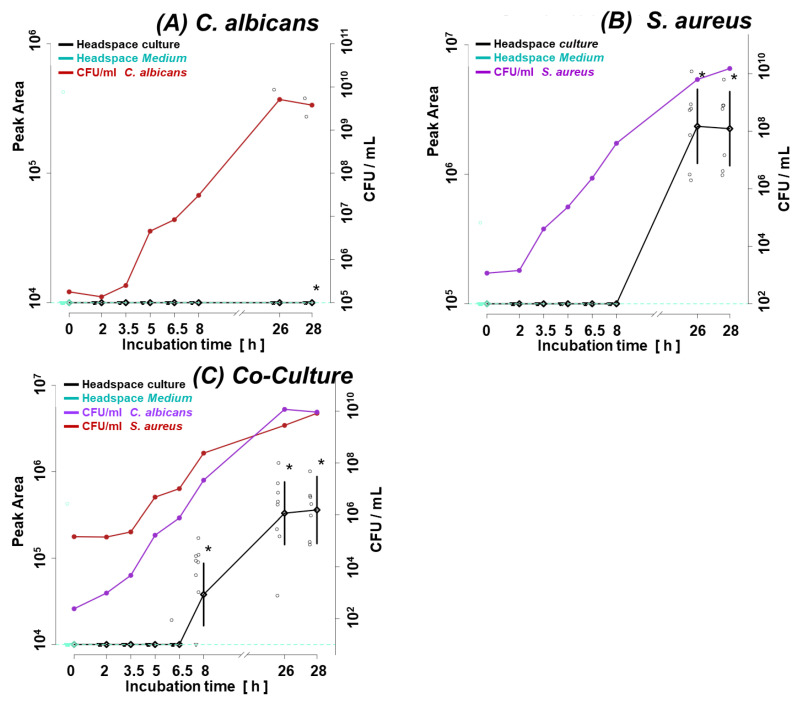
Ethyl-2-methylbutyrate (butanoic acid, 2-methyl-, ethyl ester) synthesis in the monoculture of (**A**) *C. albicans*, (**B**) *S. aureus*, and (**C**) their co-culture. Significant (*p* < 0.05) differences between VOC levels in the headspace of the culture (black line) and medium control (dotted turquoise line) are marked with a black asterisk. Circles indicate the measured metabolite abundance in the headspace at a particular time point in an individual experiment. A black line connects the median values of peak areas corresponding to the particular time point (along with the whiskers indicating the 25th and 75th percentile), while a turquoise line denotes the cut-off value, which is the median of a given VOC in the medium headspace at all time points within the experiments.

Accordingly, ethyl 2-methylbutyrate appeared as a co-culture-specific metabolite in the log phase of microbial growth since it was absent in both single cultures but present in the co-culture. However, it discriminates only in the log phase because it was produced by *S. aureus* in the steady phase in both the single and co-culture (Figure 5).

**Figure 6:**
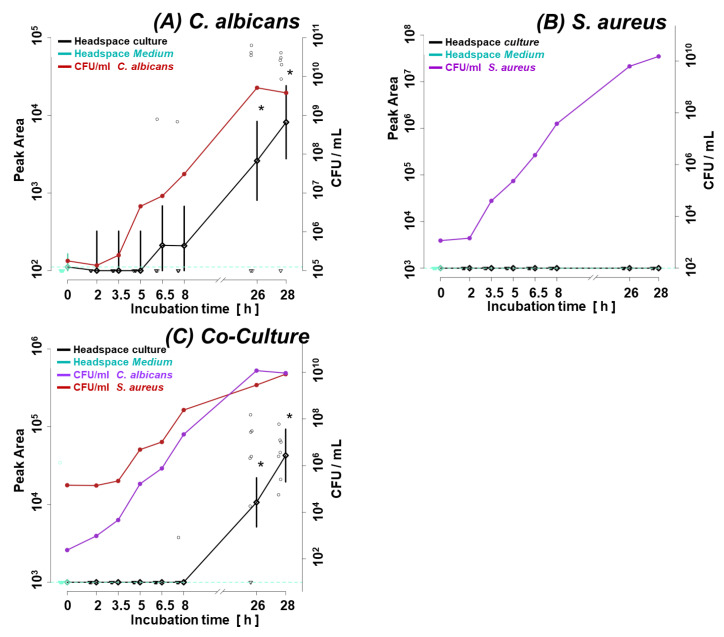
Isobutyl acetate (acetic acid, 2-methylpropyl ester) synthesis in the monoculture of (**A**) *C. albicans* and (**C**) its co-culture with *S. aureus*. Isobutyl acetate was not detected in the *S. aureus* monoculture, as presented in subfigure (**B**). Significant (*p* < 0.05) differences between VOC levels in the headspace of the culture (black line) and medium control (dotted turquoise line) are marked with a black asterisk. Circles indicate the measured metabolite abundance in the headspace at a particular time point in an individual experiment. A black line connects the median values of peak areas corresponding to the particular time point (along with the whiskers indicating the 25th and 75th percentile), while a turquoise line denotes the cut-off value, which is the median of a given VOC in the medium headspace at all time points within the experiments.

**Figure 7:**
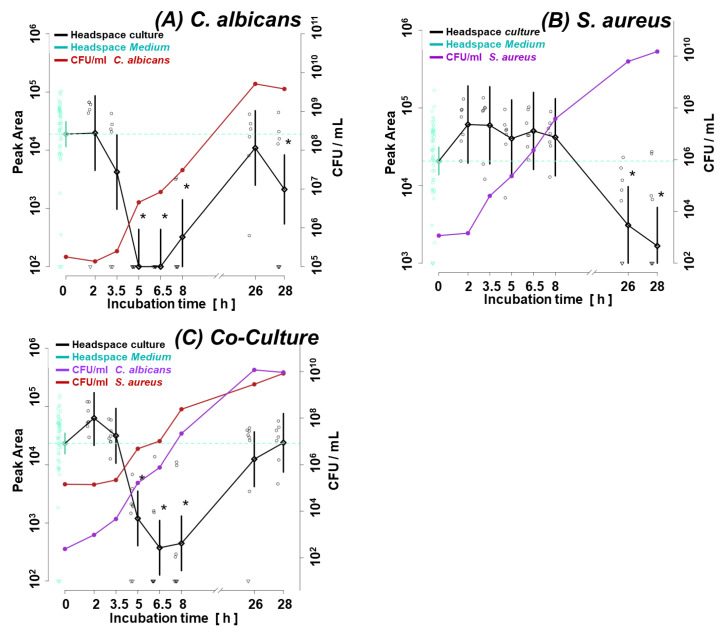
Carbon disulfide (CS_2_) synthesis in the monoculture of (**A**) *C. albicans*, (**B**) *S. aureus*, and (**C**) their co-culture. Significant (*p* < 0.05) differences between VOC levels in the headspace of the culture (black line) and medium control (dotted turquoise line) are marked with a black asterisk. Circles indicate the measured metabolite abundance in the headspace at a particular time point in an individual experiment. A black line connects the median values of peak areas corresponding to the particular time point (along with the whiskers indicating the 25th and 75th percentile), while a turquoise line denotes the cut-off value, which is the median of a given VOC in the medium headspace at all time points within the experiments.

The analogous situation, where the metabolism of a particular VOC is dominated by one of the co-cultured species, concerns isobutyl acetate (Figure 6) and carbon disulfide (Figure 7), which both were strongly related to the activity of *C. albicans* in the co-culture.

**Figure 8:**
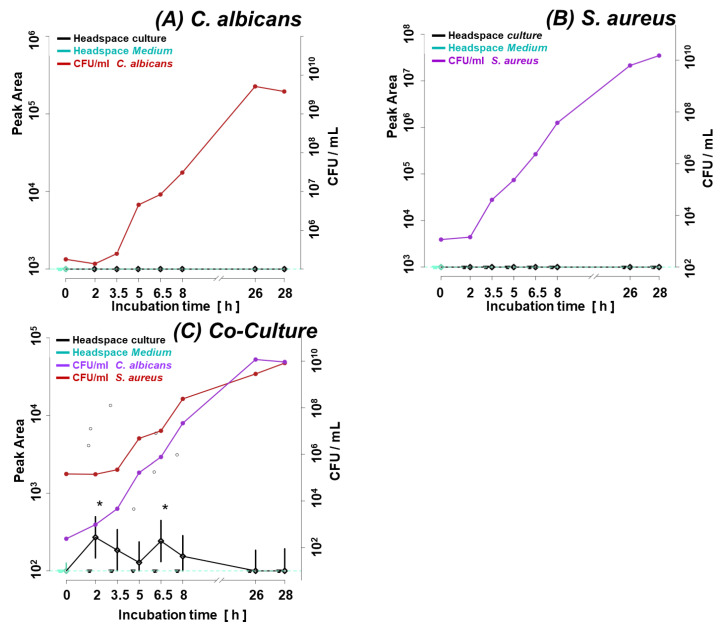
Methyl isobutyl ketone synthesis in the co-culture with *C. albicans* with *S. aureus* (**C**). Methyl isobutyl ketone was not detected in the monocultures of *C. albicans* (**A**) or *S. aureus* (**B**). Significant (*p* < 0.05) differences between VOC levels in the headspace of the culture (black line) and medium control (dotted turquoise line) are marked with a black asterisk. Circles indicate the measured metabolite abundance in the headspace at a particular time point in an individual experiment. A black line connects the median values of peak areas corresponding to the particular time point (along with the whiskers indicating the 25th and 75th percentile), while a turquoise line denotes the cut-off value, which is the median of a given VOC in the medium headspace at all time points within the experiments.

An intriguing profile was observed for methyl isobutyl ketone, which was only significantly different from that observed in the reference medium in the log phase of the co-culture (Figure 8).

**Figure 9:**
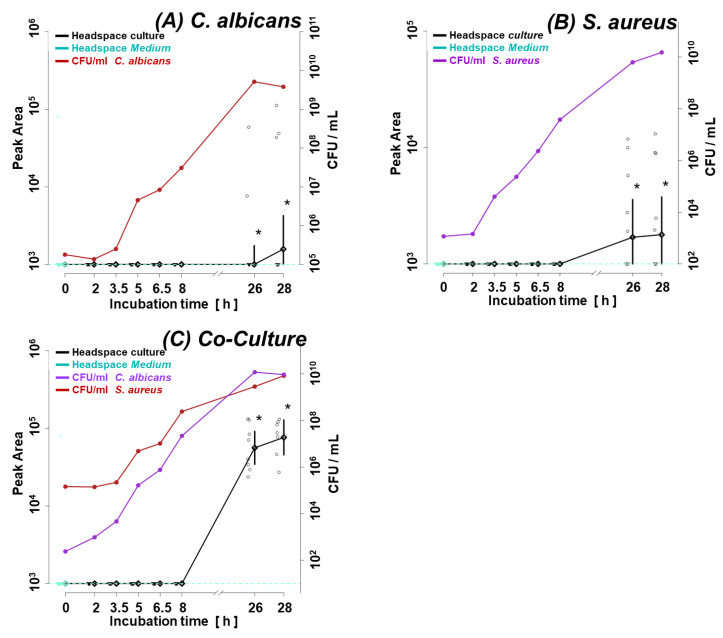
Isopentyl valerate (pentanoic acid, 3-methylbutyl ester) synthesis in the monoculture of (**A**) *C. albicans*, (**B**) *S. aureus*, and (**C**) their co-culture. Significant (*p* < 0.05) differences between VOC levels in the headspace of the culture (black line) and medium control (dotted turquoise line) are marked with a black asterisk. Circles indicate the measured metabolite abundance in the headspace at a particular time point in an individual experiment. A black line connects the median values of peak areas corresponding to the particular time point (along with the whiskers indicating the 25th and 75th percentile), while a turquoise line denotes the cut-off value, which is the median of a given VOC in the medium headspace at all time points within the experiments.

The most prominent example of metabolites reflecting synergic interactions between tested bacteria and fungi was an ester isopentyl valerate (another name: 3-methylbutyl pentanoate), whose abundance in the steady phase of the co-culture was significantly higher from both single cultures (Figure 9).

### 3.4. Bacterial Metabolites in BAL Specimens

**Figure 10:**
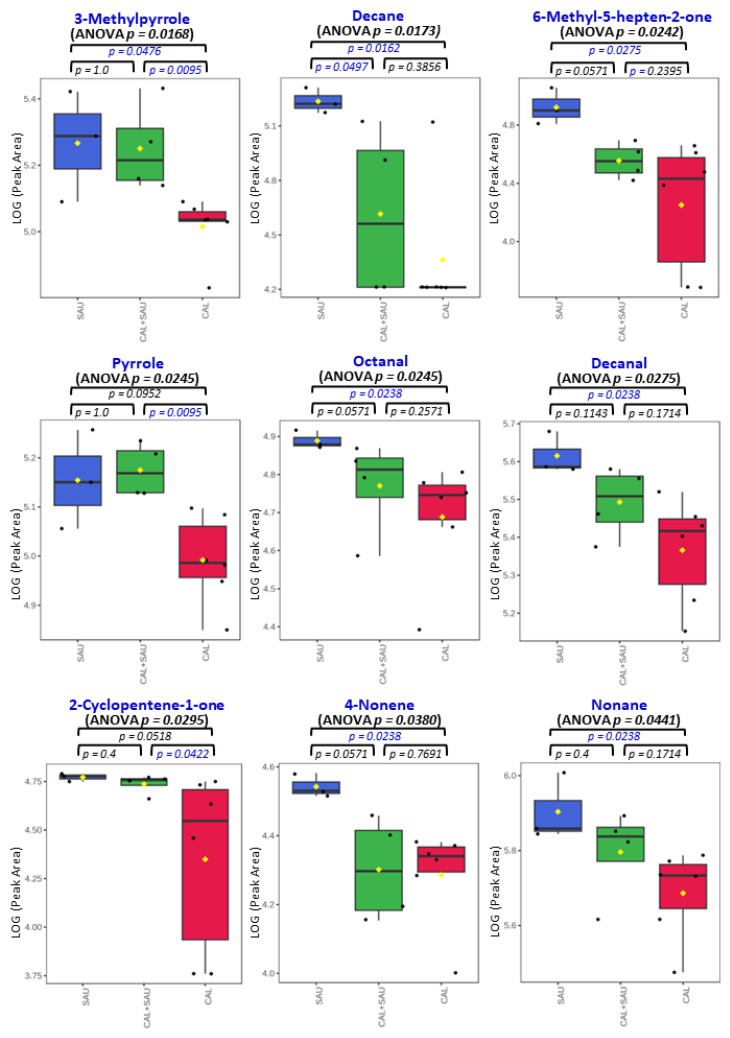
Quantities of VOCs significant (ANOVA *p* < 0.05) for comparison between pathogens found in all BAL samples isolated from VAP patients, where SAU is for a specimen with *S. aureus* (n = 3), CAL is for *C. albicans* (n = 6), and SAU+CAL is for BAL specimens with both species (n = 4). Additionally, p-values of Student’s *t*-test are given to compare two groups of interest, whereby significant differences from the non-parametric *t*-test (*p* < 0.05) are marked in blue. The black dots represent the abundance of the selected substance from all samples, whereby the whiskers indicate a 95% confidence interval around the median of each group and the yellow diamonds indicate the mean concentration of each group.

**Figure 11:**
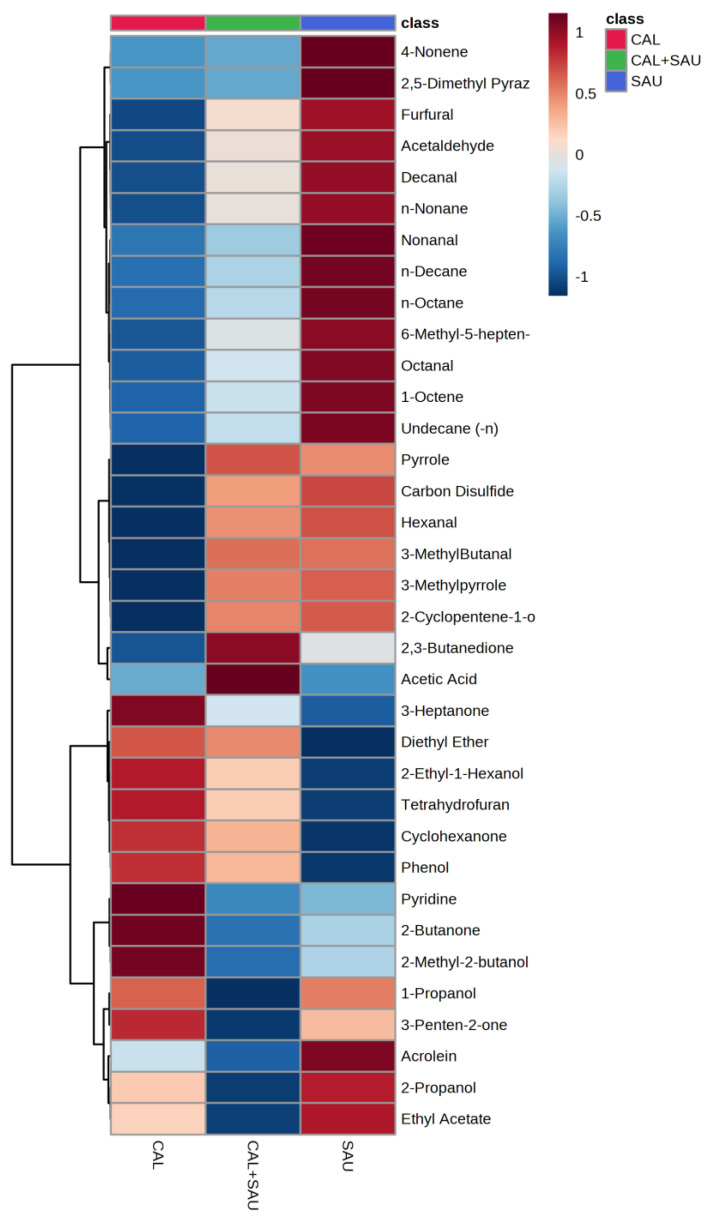
Heatmap representing averaged abundances of 35 VOCs in BAL samples for which ANOVA p-values were up to 0.25 for the comparison of all three groups of BAL specimens. Color contrast is autoscaled individually to each feature (substance) across all sample types (BAL specimens with different pathogens).

Application of the novel TFME extraction technique, optimized in our previous study [23], allows for the detection of as many as 95 VOCs in the analyzed BAL specimens. Twenty-eight of these were also synthesized by the tested microorganisms under in vitro conditions. Some microbial metabolites detected in human specimens were found at significantly different amounts in relation to the pathogen, causing pneumonia in mechanically ventilated patients. In this respect, the non-parametric ANOVA test revealed a significantly different (*p* < 0.05) abundances of nine metabolites between all three groups of BAL specimens (i.e., *S. aureus*, *C. albicans,* and the co-existence of both species), including 3-methylpyrrole, n-decane, 6-methyl-5-hepten-2-one, pyrrole, octanal, decanal, 2-cyclopenten-1-one, 4-nonene, and n-nonane (Figure 10). A narrower comparison of only two groups of the analyzed specimens with a *t*-test indicated an additional set of compounds with statistical significance (*p* < 0.05). However, given the small number of BAL samples and their high natural biological heterogeneity, the result of statistical calculations in this section should be considered in terms of a rough approximation rather than the rigid criteria for selecting biologically important VOC markers. Therefore, the data given in Table 2 comprise metabolites ordered according to the increasing p-value for the non-parametric ANOVA test of all three groups. The quantities of the first 35 VOCs with the lowest *p*-value (up to *p* = 0.25) averaged to each group of BAL specimens are given in the form of a heatmap in Figure 11. The applied higher p-value limit offers a broader overview of VOC profiles observed in infectious specimens collected from the critically ill and their more relevant comparison to the in vitro experiments.

**Figure 12:**
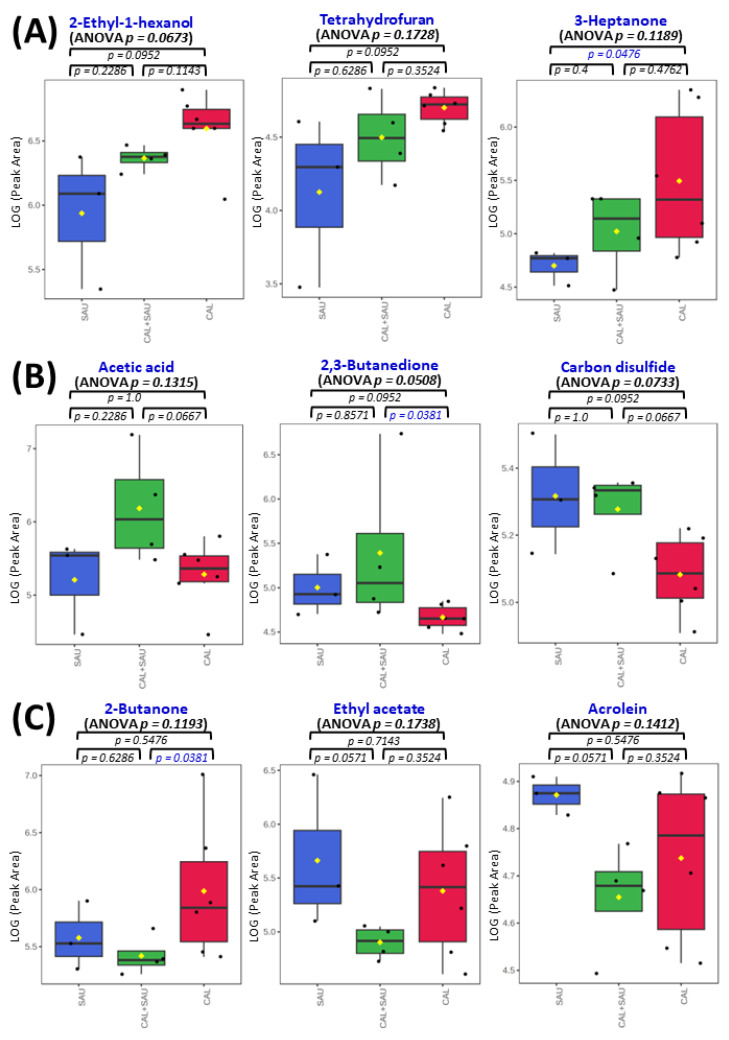
Comparison of VOC profiles in BAL specimens collected from VAP patients: (**A**) represents metabolites related to the activity of *C. albicans*; (**B**) comprises VOCs elevated in BAL specimens with both pathogens investigated; (**C**) demonstrates VOCs suppressed in BAL samples containing both studied species. ANOVA *p*-values (non-parametric) concern the comparison of all three BAL sample types together, and the remaining p-values concern the non-parametric *t*-test comparison of only two groups, indicated on plots with whiskers (statistical significance for *p* < 0.05 is marked in blue). The black dots represent the abundance of the selected substance from all samples, whereby the whiskers indicate a 95% confidence interval around the median of each group and the yellow diamonds indicate the mean concentration of each group.

The most often observed profile of VOC secretion from BAL specimens, in which both pathogens were identified, was related to the activity of *S. aureus* (as can be seen in Figure 10), but metabolites related to *C. albicans* were observed as well, e.g., 2-ethyl-1-hexanol, diethyl ether, and 3-heptanone or cyclohexanone (Figure 12A). The interactions between pathogens co-existing in BAL specimens could be observed as a synergistic effect on VOC synthesis, manifested with elevated abundances of acetic acid (Figure 12B). Intriguingly, the suppressed production of volatile metabolites such as 2-butanone, 2-propanol, and ethyl acetate (Figure 12C) was also found in co-cultures, although the growth rate of both microbes was regular. The overview of the averaged abundances of particular VOCs for each type of BAL sample is given as a heatmap in Figure 11.

## 4. Discussion

Breath gas analysis has excellent applicability for VAP diagnosis and for monitoring treatments due to its non-invasiveness, ease of acquiring serial samples, and potential to characterize underlying pathogens, allowing targeted or preemptive actions in emerging pneumonia. Breath analysis can be performed with hand-held portable sensors (reducing the costs and time), can provide rapid results, and can even be performed in real-time mode (like capnography for CO_2_ monitoring) when the direct mass spectrometric technique is applied, which could be important in a clinical setting where a timely diagnosis is critical in certain circumstances. Even unspecific information about the emerging infection, although without identification of the underlying pathogen, is still of clinical value, as it allows the intensification of clinicians’ efforts to the particular patient. This exemplifies that VOCs can complement existing diagnostic methods, including classical culturing or methods for nucleic acid amplification, such as loop-mediated isothermal amplification (LAMP) or polymerase chain reaction (PCR). While both LAMP and PCR are powerful techniques used for the rapid and specific diagnosis of bacterial infections, they require careful implementation and consideration of their limitations to ensure accurate and reliable results. Both LAMP and PCR detect DNA from live and dead bacteria, which can lead to false-positive results if non-viable bacteria are present in the sample, complicating the distinction between active infections and past infections or contamination, particularly since they are highly sensitive, and even small amounts of contaminant DNA can lead to false-positive results, similar to poorly designed primers that could lead to non-specific amplification. This necessitates rigorous contamination control procedures and careful handling of samples and reagents. On the other hand, clinical samples, such as urine, sputum, or blood, can contain substances inhibiting amplification reactions and lead to false-negative results (if not properly addressed during the sample preparation step). The standard LAMP and PCR techniques are primarily used only for the qualitative assessment of the presence of pre-selected microorganisms in the material collected from, e.g., the respiratory tract, which in mechanically ventilated patients may require invasive procedures. Moreover, in some patient groups, already after such qualitative determination of the pathogen, it is further recommended to additionally perform quantitative determination, e.g., traditional microbial culturing or DNA isolation followed by quantitative PCR from difficult-to-process and “risky” samples (that may be impossible to handle in some laboratories for a large number of patients), further increasing the costs and extending the testing time putting patients at risk of disease progression. The necessity of quantitative analysis requires additional modifications and specialized equipment, such as real-time PCR (qPCR), high-quality reagents, DNA/RNA isolation from clinical samples, and trained personnel with technical expertise to perform the entire procedure and appropriately interpret the qPCR (or LAMP) results, all of which increase the total costs. Either way, breath VOC analysis can be used as a routine and non-invasive method for rapid screening that supports clinical decisions and indicates the need for further, more specific and more laborious diagnostic tests at the early stage of emerging infection.

At the present stage, the method utilizing analysis of bacterial VOC markers in exhaled breath is not yet developed for clinical application, and further investigations to define its possible value are needed. To achieve this ambitious goal, one needs a detailed knowledge of factors affecting the synthesis of microbial VOCs, particularly in the case of multi-pathogenic infections, since microorganisms can exhibit both competitive and synergistic behavior with each other and human host cells. It was shown by Filkins et al. [27] that the presence of *P. aeruginosa* induces a change in *S. aureus* metabolism and shifts its aerobic respiration to fermentation (hence the production of lactate instead of acetate) and ultimately reduces the viability of *S. aureus*. The suppression of *S. aureus* growth, especially in its early phase, was induced by *E. coli*, as Chen et al. [20] demonstrated by monitoring species-specific VOCs. Among other species, the synergistic behavior between *P. aeruginosa* and *E. cloacae* was observed by Lawal et al. [28], where the levels of VOCs in the co-culture were significantly higher than the sum of both monocultures. The complexity of searching for volatile signatures of etiological factors (*P. aeruginosa* and respiratory syncytial virus, RSV) co-cultured with human cells (cystic fibrosis bronchial epithelial cells, CFBE) is well exemplified by Purcaro et al. [29], where a clear dominance of bacteria species over other constituents of the co-culture is shown, indisposing the differentiation of RSV-infected from non-RSV infected CFBE cells and enabling the distinction of VOCs produced by *P. aeruginosa* in the absence and presence of CFBE cells.

The working hypothesis investigated here, namely, that cross-kingdom interactions of *S. aureus* and *C. albicans* (frequently found opportunistic pathogens in VAP) influence the synthesis of microbial volatiles under in vitro conditions, is correct. Additionally, we tested the assumption of whether VOCs elucidated in vitro are convergent in the in vivo situation. To adequately address this complex problem, two different techniques were used for VOC sampling: (1) a dynamic adsorption on multibed tubes to follow the changing metabolism in growing cultures and (2) a static extraction on thin-film meshes to collect analytes ex vivo in BAL specimens from VAP patients. The latter technique enables sensitive GC-MS analysis, even from a small quantity of the sample (infectious human material) without the need for external carrier gas flow through the headspace. Compared to other static extraction techniques, it offers an enhanced adsorption efficiency and simultaneously low bleed and background due to a substantially increased stationary phase surface area. Another benefit of TFME meshes is the use of a new type of HLB adsorbent (unavailable for other commercial adsorptive techniques used in GC-MS) obtained by incorporating N-vinylpyrrolidone groups into the divinylbenzene polymer. Such a modification improves the extraction efficiency of oxygenated VOCs (i.e., aldehydes, ketones, and alcohols) due to stronger π-π interactions between adsorbent particles and electronegative elements constituting the functional groups of an analyte. The application of the TFME technique enabled precise and sensitive analysis of a wide range of VOCs in complex matrices, and the protocol applied for data evaluation allowed for an unambiguous identification of detected analytes. Importantly, both extraction techniques used in this study adsorb a wide range of VOCs, and there is no selectivity with respect to any chemical class of detected compounds.

### 4.1. In Vitro Experiments with Microbial Cultures

The results of the in vitro experiments testify that the proliferation rate of *C. albicans* and *S. aureus* under applied settings do not differ in monocultures compared to a co-cultivation of both species. The performed TD-GC-MS analyses suggest that each species activates partly different metabolic pathways in the subsequent growth phases, resulting in a dynamically changing quantity and portfolio of VOCs secreted over time. It can be well exemplified by carbon disulfide (CS_2_), which the *C. albicans* strain continuously takes up as an energy or substrate source during the entire log phase. Once the microbes enter the steady phase, their metabolism slows down, and demand for carbon disulfide reduces; hence, its level in the culture headspace rises (Figure 7A). On the contrary, *S. aureus* cultures do not consume carbon disulfide in the logarithmic phase and even seem to release this metabolite, although the difference compared to its content in the TSB medium headspace was statistically not significant (*p* = 0.924). A decrease in the CS_2_ level in *S. aureus* cultures was only observed at the latest times of cultivation (Figure 7B). Generally, it was frequently observed that the synthesis rate of certain metabolites was the highest when the microbial division was at its biological maximum (i.e., the second part of the logarithmic phase) and was substantially reduced later. In this regard, twenty-one VOCs exhibited a temporary maximum in the log phase of *C. albicans* growth with diminished abundances afterward, while only fourteen VOCs reached the highest levels in their steady phase. On the contrary, the metabolism of *S. aureus* seems slower under applied conditions, as only 11 volatiles gained a temporary maximum in the log phase. Subsequently, the secretion of metabolites increased proportionally to the total burden of *S. aureus*, reaching 24 compounds with their highest amounts in a steady stage. In turn, the number of VOCs produced in the co-cultures was nearly equally distributed between both proliferation phases, with 18 analytes higher in the log and 19 in the steady stage.

The most interesting metabolites in terms of bacterial–fungal interaction (in a setup used in this study) can be split into two groups. The first group comprises VOCs with profiles related to one microorganism but modified by the presence of another microbe. This is exemplified by carbon disulfide, which is clearly linked with the metabolic activity of the investigated fungi, as it has the same profile in *C. albicans* cultured alone and mixed with *S. aureus* (compare Figure 7A,C). However, during the log phase, the abundance of carbon disulfide was significantly higher in the co-culture compared to sole *C. albicans* (Figure 2), which can be only partly explained by the weak (statistically insignificant) production of this metabolite by *S. aureus* (Figure 7B). Apparently, the catabolism of carbon disulfide by *C. albicans* was temporarily weaker due to the presence of bacteria. The altered metabolic activity of *C. albicans* in the co-culture seems to be also confirmed by isobutyl acetate, whose first release was observed after 6.5 h in monocultures of *C. albicans* but not until 25 h in the co-culture. Intriguingly, despite the delayed synthesis, the ultimate abundance of isobutyl acetate was significantly higher in the mixed compared to the monoculture of *C. albicans*. Analogously, the existence of ethyl 2-methylbutyrate in the co-culture is related to the metabolic activity of *S. aureus*, as it was never observed in a single culture of *C. albicans* in the present study (Figure 5A). However, the inter-species interactions lead to its much faster synthesis by *S. aureus* when it grows in the co-culture compared to its monoculture (synthesis within 8 h instead of 25 h after inoculation). Nonetheless, there are no significant differences in the synthesis of this metabolite once the maximum density of bacteria is reached between sole and mixed cultures (Figure 5 B,C).

The second group comprises volatile metabolites, whose quantities significantly increase in co-cultures due to the synergism in bacterial–fungal interactions. This is exemplified by isopentyl valerate, whose abundance increases 3-fold in the steady phase of the co-culture compared to the sum of its amounts in both single cultures. Therefore, an increase in isopentyl valerate synthesis during the steady phase of the co-culture cannot be explained by the cumulative effect summarizing its production by each species alone (Figure 9A–C). A similar situation concerns the release of methyl isobutyl ketone. This metabolite was only detected during the log phase in the co-culture (from 2 h to 6.5 h), after which it was no longer synthesized. Noticeably, methyl isobutyl ketone was not observed in the headspace of single cultures of both species tested in this study.

### 4.2. Ex Vivo Analysis of Human Specimens

In the second part of our work, the bronchoalveolar lavage specimens were collected from mechanically ventilated patients known to be infected explicitly by the sole *C. albicans*, sole *S. aureus,* or both species together. Due to the unexpectedly low number of BAL specimens (available temporarily in the local hospital participating in our study) that fulfilled our inclusion criteria, the results gathered should be considered in terms of a rough comparison of volatiles synthesized by tested pathogens under in vitro (model study) and in vivo (clinical samples) conditions and not in terms of selection of species-specific biomarkers for pathogen identification in clinical settings.

One of the most interesting observations in this comparison is the noticeably higher influence of *S. aureus* on the overall VOC portfolio in BAL samples, while the *C. albicans* strain determined the metabolite range in the in vitro cultures. In this regard, the synthesis of all aldehydes and hydrocarbons was associated with the presence of *S. aureus* in the BAL specimens collected from critically ill patients, regardless of whether it was a sole pathogen in the sample or co-existing with *C. albicans* (Figure 10). These results are in agreement with the previous studies reporting aldehydes (with 3-methylbutanal being particularly abundant) as an important group of volatile metabolites synthesized by *S. aureus* [25,30]. Another example is carbon disulfide, which was observed at elevated levels when *S. aureus* was present in both human specimens and co-cultures. On the other hand, the activity of *Candida albicans* in BAL specimens determined the excretion of certain alcohols and ketones, including 2-ethyl-1-hexanol, 2-methyl-2-butanol, 3-heptanone, or cyclohexanone (Table 2). Importantly, none of these compounds was metabolized under in vitro conditions by the pathogens tested in this study. There is a curious case of 3-heptanone, as this ketone was only observed in BAL samples, whereas 4-heptanone was present in the in vitro cultures of these fungi (significantly released during a log phase of mono- and co-cultures). Since both the mass spectrum and the retention time from the chromatographic separation are different for these two compounds, there is no risk of mistaking them. Furthermore, 4-heptanone was previously reported in the pilot clinical study on pathogen identification using breath gas analysis in VAP patients infected with *Candida albicans* [13]. The reason for the discrepancy between 3-heptanone in biological material and 4-heptanone in cultures is unknown.

The interactions between pathogens co-existing in BAL specimens were also observed, as their synergistic effect on VOC synthesis manifested with elevated abundances of acetic acid (Figure 12B). This compound was synthesized under in vitro conditions in small quantities by both *C. albicans* in its log phase and by *S. aureus* in its steady growth phase. Still, the levels of acetic acid in co-cultures were lower than in both monocultures, whereas in BAL, they reached the highest amounts in specimens containing both pathogens. In contrast, volatile metabolites such as 2-butanone, acrolein, and ethyl acetate were suppressed in BAL samples with mixed pathogens (Figure 12C). Amongst them, the synthesis of ethyl acetate was directly proportional and acrolein was inversely proportional to the proliferation rate of *C. albicans* in mono- and co-cultures.

### 4.3. Comparison of the In Vitro and In Vivo Metabolome

Consistent and inconsistent results between in vitro and ex vivo/in vivo experiments are important because they demonstrate the complexity of volatilome studies and the need for further research, especially with human materials (BAL, blood, etc.).

Overall, the presented data show that the co-existence of both species under in vitro conditions does not affect their proliferation rate compared to monocultures, but it does affect the synthesis of their volatile metabolites. Under optimal growth conditions, *C. albicans* dominates the volatilome in the co-cultures. In BAL material, S. aureus seems to have benefited from its co-existence with *C. albicans* (Figure 10), and it adjusts the portfolio of VOCs to the new metabolic conditions in a far more complex matrix. *S. aureus* enhances the synthesis of new compounds, particularly hydrocarbons and aldehydes, while suppressing the production of esters (compared to in vitro conditions). Most of the produced VOCs are associated with *S. aureus* in this situation. The reason why some metabolites exhibit distinct profiles in culture and biological material remains unknown. Still, the co-existence of both tested species in the biological sample yields a particular volatile metabolic signature, with acetic acid, methyl methacrylate, and carbon disulfide being the most prominent examples.

Additional studies with other relevant pathogens should be performed to further investigate the inter-species interactions and their impact on the synthesis of volatile metabolites. For clinical relevance in pneumonia diagnosis, the focus should be on the analysis of the most prevalent pathogens’ combinations with additional clinical impact on ICU patients. An example of this is an investigation of possible antibiotic treatment with its implied changes in pathogen metabolism. In addition to in vitro and ex vivo studies, breath gas analysis of ICU patients with confirmed coinfections (with the pathogens of interest) should be compared to the present in vitro study.

### 4.4. Study Limitation

The most significant limitation of this type of research is always the availability of clinical samples, especially if they are to be additionally divided, e.g., according to the etiological factor to address the translation of in vitro findings into in vivo settings; hence, even a small number of samples in our work is still an advantage. Despite the newest available data from the NHSN report indicating *S. aureus* to be the most dominant pathogen in VAP patients (29.6% of cases in the USA) [1], its prevalence in the ICU ward participating in our study (Bydgoszcz, Poland) was considerably lower (9.8% amongst all 92 BAL samples analyzed with TFME-GC-MS). Therefore, the small number of BAL specimens analyzed, which fulfill the inclusion criteria—either sole *C. albicans* or *S. aureus* or the presence of both—together with the variability of clinical conditions of VAP patients, potentially affect the composition of the isolated BAL specimens, which can be considered a limitation of our work. Another factor potentially affecting the comparison of VOCs found in the cultures and BAL specimens is the growth environment, whereby the TSB medium, however relatively poor in nutrients, certainly offers more optimal conditions for the proliferation of microorganisms. Nevertheless, the distinction between these two different environments is not a drawback of our study but rather a necessary aspect to consider in real-world applications. Despite these aspects, the key aims of this work have been achieved, namely, that the volatile metabolites released by the tested microorganisms in single- and co-cultures were identified, their dynamically changing profiles were recognized, and part of them was further found in biological material isolated from patients infected with these pathogens, demonstrating the suitability of a novel microextraction technique for analysis of clinical samples.

### 4.5. Concluding Remarks

To determine whether putative interactions between *S. aureus* and *C. albicans* affect the secretion of VOCs under in vitro conditions, the solid phase extraction on multibed sorption tubes was used for sample collection and VOC preconcentration before GC-MS analysis. To collect analytes ex vivo in BAL specimens from VAP patients, the static extraction on thin-film meshes coated with a novel hydrophilic–lipophilic-balanced adsorbent was used. Both analytical protocols proved well suited for the chosen application, allowing the precise and reproducible analysis of the microbial volatilome.

The gathered data demonstrated the importance of considering the growth phase in which bacteria sampling is performed when comparing the volatilomes of certain strains determined in unrelated studies. In this regard, one must pay particular attention to selecting “biomarkers” for pathogen identification, especially if applied to clinical settings with all putative consequences, such as treatment choice. It is noticeable that most of the VOCs in the co-culture are associated with *C. albicans* metabolism, and some of them exclusively originate from this yeast (e.g., isobutyl acetate), while considerably fewer VOCs are released from *S. aureus,* mainly in the latest phase of its growth (e.g., ethyl-2-methylbutyrate). Even more interesting are compounds, such as methyl isobutyl ketone, which exhibit no difference from the control medium in a single culture but significantly differ in the co-culture compared to the control medium. In the BAL material collected from VAP patients, *S. aureus* seems to adjust the portfolio of VOCs to the new metabolic conditions and enhances the synthesis of new compounds, particularly hydrocarbons and aldehydes, while suppressing the production of esters and alcohols (compared to in vitro conditions). The co-existence of both tested species in the biological sample yields a volatile metabolic signature, including acetic acid, methyl methacrylate, and carbon disulfide. Altogether, these observations give molecular evidence of the presence of the respective single microbe on the one hand and suspect potential multi-pathogenic infections on the other hand. The reported microbial metabolites should be used to pursue non-invasive VAP diagnosis in future clinical studies.

Our preliminary research highlights the potential for VOC profiles to reveal unique metabolic interactions between co-existing pathogens. This complexity, rather than being a drawback, could offer more nuanced insights, as identifying VOC signatures specific to co-infections may lead to the development of a more precise diagnostic tool that is robust against the variability introduced by different microbial environments in future studies.

## Figures and Tables

**Table 1:** List of compounds identified in the investigated microbial cultures. “↑” indicates the significantly (*p* < 0.05) increased and “↓” indicates decreased levels in the culture compared to the reference medium, and the period of the growth phase during which significant change was observed is given in brackets. CAS is a substance-unique number in Chemical Abstract Service, tR is the retention time [min] of a substance in a given GC program, the target ion is an extracted ion used for the integration of a chromatographic peak area, reference ions are used to additionally confirm substance identification during integration, CAL stands for *Candida albicans* cultures, SAU for *Staphylococcus aureus*, and the co-culture is a mix of both microorganisms.

Class	No.	Compound Name	Common Name	CAS	t_R_ [min]	Target Ion [*m*/*z*]	Reference Ions [*m*/*z*]	CAL	SAU	Co-Culture
**VOCs with confirmed retention times of reference standards**										
Alcohols	1	Methyl alcohol	Methanol	67-56-1	7.66	31	32, 29	↑ (log)	↑ (steady)	↑ (log)
	2	Ethyl alcohol	Ethanol	64-17-5	12.29	31	45, 46, 43	↑ (log)	↑ (steady)	↑ (log) ↓ (steady)
	3	2-Propanol	Isopropanol	67-63-0	18.03	45	43, 41, 59	↑ (log, steady)		↑ (log, steady)
	4	*n*-Propyl alcohol	1-Propanol	71-23-8	22.30	31	60, 59, 27	↑ (log)	↑ (steady)	↑ (log)
	5	2-Propanol, 2-methyl-	*tert*-Butanol	75-65-0	25.61	59	57, 60, 51	↑ (log, steady)	↑ (steady)	↑ (log, steady)
	6	1-Propanol, 2-methyl-	Isobutanol	78-83-1	31.66	41	43, 74, 31	↑ (log, steady)	↑ (steady)	↑ (log, steady)
	7	*n*-Butyl alcohol	1-Butanol	71-36-3	43.76	56	31, 41, 55	↑ (log)	↑ (steady)	↑ (log)
	8	1-Butanol, 3-methyl-	Isopentanol	123-51-3	45.40	55	70, 42, 41	↑ (log, steady)	↑ (steady)	↑ (log, steady)
Aldehydes	9	Acetic aldehyde	Acetaldehyde	75-07-0	8.06	29	43, 44, 42	↑ (log)	↑ (steady)	↑ (log) ↓ (steady)
	10	2-Propenal	Acrolein	107-02-8	13.95	27	56, 55, 53			↓ (log)
	11	Propanal, 2-methyl-	Isobutanal	78-84-2	24.69	72	41, 43, 39		↓ (steady)	
	12	Butyrylaldehyde	Butanal	123-72-8	27.29	44	57, 72, 39	↓ (log, steady)		↓ (log, steady)
	13	Butanal, 3-methyl-	Isovaleraldehyde	590-86-3	36.58	44	58, 71, 86	↓ (steady)		↓ (steady)
	14	2-Butenal, 3-methyl-	3-methylcrotonaldehyde	107-86-8	47.04	84	83, 55, 39	↓ (steady)		↓ (steady)
	15	Benzoic aldehyde	Benzaldehyde	100-52-7	64.48	106	105, 77, 51	↓ (log, steady)	↓ (steady)	↓ (log, steady)
Acids	16	Acetic acid		64-19-7	32.94	60	45, 43, 42	↑ (log)	↑ (steady)	↑ (log)
	17	Hexanoic acid	Caproic acid	142-62-1	59.10	60	73, 87, 41	↑ (steady)	↑ (steady)	↑ (steady)
Esters	18	Formic acid, ethyl ester	Ethyl formate	109-94-4	17.51	74	45, 31, 43	↑ (log)	↑ (log, steady)	↑ (log)
	19	Acetic acid, methyl ester	Methyl acetate	79-20-9	18.19	43	74, 59		↑ (steady)	
	20	Acetic acid, ethyl ester	Ethyl acetate	141-78-6	29.47	43	70, 61, 45	↑ (log, steady)	↑ (steady)	↑ (log, steady)
	21	Acetic acid, propyl ester	*n*-Propyl acetate	109-60-4	42.09	61	73, 43, 27		↑ (steady)	
	22	Acetic acid, 2-methylpropyl ester	Isobutyl acetate	110-19-0	50.85	73	56, 43, 86	↑ (steady)		↑ (steady)
	23	Acetic acid, butyl ester	*n*-Butyl acetate	123-86-4	53.09	43	56, 73, 61		↑ (steady)	
	24	Ethyl, 2-methylbutyrate		7452-79-1	57.63	57	102, 85, 74	↑ (steady)	↑ (steady)	↑ (log, steady)
	25	Ethyl, 3-methylbutyrate	Ethyl isovalerate	108-64-5	58.20	88	85, 60, 57	↑ (steady)	↑ (steady)	↑ (steady)
	26	1-Butanol, 3-methyl-, acetate	Isoamyl acetate	123-92-2	59.33	70	61, 55	↑ (log, steady)	↑ (steady)	↑ (log, steady)
Ketones	27	Propanone	Acetone	67-64-1	15.08	43	58, 57, 44		↓ (steady)	↑ (log)
	28	2-Pentanone	Methyl propyl ketone	107-87-9	39.89	86	43, 57, 71	↑ (log, steady)	↑ (log)	↑ (log, steady)
	29	4-heptanone	Dipropyl ketone	123-19-3	58.60	114	71, 43, 41	↑ (log)		↑ (log)
	30	2-Heptanone	Amyl methyl ketone	110-43-0	59.87	58	43, 71, 114	↑ (log, steady)		↑ (log, steady)
	31	3-Octanone	Amyl ethyl ketone	106-68-3	67.09	99	128, 72, 57	↑ (log, steady)		↑ (steady)
	32	2-Nonanone	Heptyl methyl ketone	821-55-6	72.08	58	43, 71, 142	↑ (log, steady)		↑ (log, steady)
VSCs	33	Carbon disulfide	CS2	75-15-0	15.79	76	44, 78, 38	↓ (log)	↓ (steady)	↓ (log)
	34	Disulfide, dimethyl	DMDS	624-92-0	42.02	94	43, 79, 61	↓ (steady)		↓ (log, steady)
	35	2-Acetylthiazole		24295-03-2	64.48	99	127, 112, 58	↓ (log, steady)		↓ (steady)
**VOCs identified with Mass Spectra Library Match (not confirmed with standards)**										
Alcohols	36	2-Butanol	*sec*-Butanol	78-92-2	31.31	45	59, 41	↑ (log, steady)		↑ (log, steady)
	37	1-Butanol, 2-methyl-	sec-butylcarbinol	137-32-6	46.97	57	56, 29, 41	↑ (log, steady)		↑ (log, steady)
Aldehydes	38	Pentanal	Valeraldehyde	110-62-3	40.13	44	58, 86, 41	↓ (steady)		↓ (steady)
	39	2-Butenal, 2-methyl-	2-Methylcrotonaldehyde	1115-11-3	43.27	84	55, 39, 41	↓ (log, steady)	↓ (steady)	↓ (log, steady)
	40	2-Furaldehyde	Furfural	98-01-1	49.39	96	95, 67, 39	↓ (steady)	↓ (steady)	↓ (steady)
	41	Hexanal	Caproaldehyde	66-25-1	52.23	56	72, 82, 44			↓ (log, steady)
Acid	42	Propionic acid, 2-methyl-	Isobutyric acid	79-31-2	52.75	43	73, 41, 27	↑ (log)	↑ (steady)	↑ (steady)
Esters	43	Formic acid, butyl ester	*n*-Butyl formate	592-84-7	35.10	56	31, 41, 73		↑ (steady)	
	44	Butanoic acid, ethyl ester	Ethyl *n*-butyrate	105-54-4	52.23	71	88, 43, 60	↑ (log)	↑ (steady)	↑ (log)
	45	Pentanoic acid, 3-methylbutyl ester	Isoamyl valerate	2050-09-1	73.41	85	70, 55, 43	↑ (steady)	↑ (steady)	↑ (steady)
Ketones	46	2-Butanone	Ethyl methyl ketone	78-93-3	27.66	72	43, 57, 42	↑ (log)	↑ (log)	↑ (log)
	47	2,3-Pentanedione	Acetylpropionyl	600-14-6	41.10	29	43, 57, 100	↓ (log, steady)	↑ (log) ↓ (steady)	↓ (log, steady)
	48	2-Butanone, 3-hydroxy	Acetoin	513-86-0	43.12	45	43, 88		↑ (steady)	
	49	2-Pentanone, 4-methyl-	Isobutyl methyl ketone	108-40-1	48.45	43	58, 85, 100			↑ (log)
	50	3-Hexanone, 2-methyl-	Isopropyl propyl ketone	7379-12-6	58.8	71	114, 43, 41	↑ (log)		↑ (log)
Hyrocarbons	51	Propane, 2-methyl-	Isobutane	75-28-5	9.91	43	41, 27, 57	↑ (log, steady)		↑ (log, steady)
	52	1-Propene, 2-methyl-	Isobutene	115-11-7	10.36	41	56, 39, 27	↓ (log)		
	53	1-Butene		106-98-9	11.39	41	56, 39, 27	↓ (log) ↑ (steady)		↓ (log) ↑ (steady)
	54	2-Pentene	2-Pentene	109-68-2	19.63	55	70, 53, 56			↑ (steady)
	55	2-Butene, 2-methyl-	Amylene	513-35-9	21.64	55	70, 41, 39			↑ (steady)
	56	1-Pentene, 3-methyl-	3-methyl-1-pentene	760-20-3	43.63	55	69, 84, 41	↑ (log)		
VSCs	57	Sulfur dioxide	Sulfur dioxide	2025884	7.90	64	48		↑ (steady)	
	58	Methanethiol	Mercaptomethane	74-93-1	8.87	47	48, 45			↓ (steady)
	59	1-Mercapto-2-propanone	Mercaptoacetone	24653-75-6	36.12	90	43, 45, 47	↑ (steady)	↓ (log) ↑ (steady)	↑ (steady)
VNCs	60	Methyl cyanide	Acetonitrile	75-05-8	12.45	41	40, 39, 38	↓ (log)	↓ (steady)	↓ (log)
	61	Propanenitrile, 2-methyl-	Isobutyronitrile	78-82-0	30.53	42	68, 54, 41			↑ (log)
	62	Butanenitrile, 3-methyl	Isoamylnitrile	625-28-5	44.52	43	41, 68, 27	↓ (steady)	↓ (steady)	↓ (steady)
	63	Acetamide, N,N-dimethyl	DMA	127-19-5	53.78	87	72, 44, 43	↑ (log)	↑ (steady)	↑ (log)
	64	Pyrazine, 2,6-dimethyl-		108-50-9	57.61	42	108, 40, 67			↑ (log, steady)
Others	65	Furan	Furan	110-00-9	14.20	68	39, 38, 40	↓ (log)	↓ (steady)	↓ (log)
	66	Propane, 2-methoxy-2-methyl-	*tert*-Butyl methyl ether	1634-04-4	28.97	73	57, 41, 29	↑ (log)		↑ (log)
	67	Toluene		108-88-3	47.44	91	92, 65, 39			↓ (steady)
	68	Oxazole, 2,4,5-trimethyl	Trimethyloxazole	20662-84-4	53.50	111	68, 55, 82	↓ (steady)	↑ (log)	↓ (steady)

**Table 2:** List of compounds identified in the BAL specimens isolated from VAP patients. Compounds identified based on only Mass Spectra Library match (NIST 2018), i.e., without confirmation of the retention time of the pure standard, are given in italics and labeled with an asterisk. Mean peak areas of respective substances within a given sample type are given along with the *p*-values for statistical comparison of VOC abundances, whereby the ANOVA concerns a non-parametric test to compare all three sample types together. The remaining *p*-values concern the *t*-test, comparing only two groups of interest (significant *p* < 0.05 are given in blue bold font). CAS is a substance-unique number in Chemical Abstract Service, t_R_ is the retention time [min] of a substance in a given GC program, the target ion is an extracted ion used for the integration of a chromatographic peak area, reference ions are used to additionally confirm substance identification during integration, CAL stands for BAL specimens with *Candida albicans*, SAU for BAL with *Staphylococcus aureus*, and MIX denotes BAL specimens with both pathogens present. Compounds are arranged according to the increasing *p*-value of the ANOVA test.

Compound Name	CAS	t_R_ [min]	Target Ion [*m*/*z*]	Reference Ion [*m*/*z*]	Mean PA (MIX)	Mean PA (SAU)	Mean PA (CAL)	*p*-Value (ANOVA)	*p*-Value (SAU-CAL)	*p*-Value (SAU-MIX)	*p*-Value (CAL-MIX)
3-Methylpyrrole	616-43-4	54.360	80	44, 43, 42	1.85 × 10^5^	1.94 × 10^5^	1.05 × 10^5^	**0.016774**	**0.047619**	1	**0.0095238**
n-Decane	124-18-5	71.400	57	43, 71, 142	5.38 × 10^4^	1.73 × 10^5^	2.21 × 10^4^	**0.017276**	**0.01621**	**0.049746**	0.35857
6-Methyl-5-hepten-2-one	110-93-0	69.140	108	69, 55, 93	3.70 × 10^4^	8.59 × 10^4^	2.35 × 10^4^	**0.024258**	**0.027532**	0.057143	0.23953
Octanal	124-13-0	70.320	84	69, 56, 100	6.07 × 10^4^	7.75 × 10^4^	5.10 × 10^4^	**0.024507**	**0.02381**	0.057143	0.25714
Pyrrole	109-97-7	44.650	67	39, 41, 40	1.50 × 10^5^	1.45 × 10^5^	9.99 × 10^4^	**0.024507**	0.095238	1	**0.0095238**
Decanal	112-31-2	76.010	57	43, 55, 82	3.16 × 10^5^	4.15 × 10^5^	2.42 × 10^5^	**0.027504**	**0.02381**	0.11429	0.17143
2-Cyclopentene-1-one	930-30-3	53.085	82	39, 53, 54	5.48 × 10^4^	5.92 × 10^4^	3.03 × 10^4^	**0.029498**	0.051822	0.4	**0.042197**
* 4-Nonene	*10405-85-3*	*66.105*	*126*	*69*, *56, 41*	*2.09 × 10^4^*	*3.49 × 10^4^*	*2.01 × 10^4^*	***0.038036***	***0.02381***	*0.057143*	*0.7619*
n-Nonane	111-84-2	66.600	57	43, 85, 99	6.43 × 10^5^	8.13 × 10^5^	5.00 × 10^5^	**0.044118**	**0.02381**	0.4	0.17143
2,3-Butanedione	431-03-8	31.663	86	43, 42, 41	1.44 × 10^6^	1.24 × 10^5^	4.86 × 10^4^	**0.050754**	0.095238	0.85714	**0.038095**
2-Ethyl-1-Hexanol	104-76-7	71.325	57	70, 83, 41	2.36 × 10^6^	1.20 × 10^6^	4.58 × 10^6^	0.067353	0.095238	0.22857	0.11429
* Diethyl Ether	*60-29-7*	*24.505*	*74*	*45, 59, 41*	*5.61 × 10^3^*	*0*	*4.63 × 10^3^*	*0.068751*	***0.025601***	*0.11867*	*0.91429*
Carbon Disulfide	75-15-0	20.163	76	44, 78, 38	1.95 × 10^5^	2.19 × 10^5^	1.25 × 10^5^	0.073341	0.095238	1	0.066667
3-MethylButanal	590-86-3	42.417	58	71, 41, 86	4.82 × 10^6^	4.98 × 10^5^	1.23 × 10^5^	0.080522	**0.02381**	0.4	0.47619
n-Octane	111-65-9	59.243	85	114, 43, 57	2.56 × 10^5^	3.24 × 10^5^	2.28 × 10^5^	0.11477	0.095238	0.11429	0.60952
3-Heptanone	106-35-4	62.400	57	85, 72, 114	1.37 × 10^5^	5.25 × 10^4^	7.91 × 10^5^	0.11894	**0.047619**	0.4	0.47619
2-Butanone	78-93-3	31.539	72	43, 57, 42	2.79 × 10^5^	4.46 × 10^5^	2.41 × 10^6^	0.11927	0.54762	0.62857	**0.038095**
Acetic Acid	64-19-7	36.000	60	45, 43, 42	4.66 × 10^6^	2.57 × 10^5^	2.69 × 10^5^	0.13148	1	0.22857	0.066667
Cyclohexanone	108-94-1	60.235	98	69, 42, 83	1.51 × 10^6^	6.74 × 10^5^	1.77 × 10^6^	0.13608	**0.047619**	0.4	0.60952
2-Propanol	67-63-0	22.209	45	43, 41, 59	1.42 × 10^7^	1.40 × 10^8^	8.60 × 10^7^	0.13834	0.54762	0.11429	0.17143
Acrolein	107-02-8	17.158	56	55, 53, 37	4.63 × 10^4^	7.46 × 10^4^	5.83 × 10^4^	0.14818	0.54762	0.057143	0.35238
Nonanal	124-19-6	73.300	98	70, 43, 56	1.19 × 10^5^	1.66 × 10^5^	1.06 × 10^5^	0.15147	0.095238	0.22857	0.60952
* 2-Methyl-2-butanol	*75-85-4*	*43.365*	*73*	*59, 55, 43*	*1.72 × 10^5^*	*3.00 × 10^5^*	*7.65 × 10^5^*	*0.16668*	*0.2619*	*0.85445*	*0.10874*
* 2,5-Dimethyl Pyrazine	*123-32-0*	*61.766*	*108*	*42, 81, 39*	*5.06 × 10^4^*	*2.49 × 10^5^*	*5.10 × 10^4^*	*0.1714*	*0.16667*	*0.11429*	*0.91429*
* Tetrahydrofuran	*109-99-9*	*30.900*	*71*	*72, 42, 41*	*3.67 × 10^4^*	*2.00 × 10^4^*	*5.16 × 10^4^*	*0.17282*	*0.095238*	*0.62857*	*0.35238*
* 1-Octene	*111-66-0*	*58.528*	*55*	*70, 41, 83*	*2.76 × 10^5^*	*3.66 × 10^5^*	*2.45 × 10^5^*	*0.17282*	*0.16667*	*0.11429*	*0.7619*
Furfural	98-01-1	53.615	96	95, 67, 39	7.93 × 10^4^	9.43 × 10^4^	6.31 × 10^4^	0.17282	0.16667	0.4	0.25714
Ethyl Acetate	141-78-6	33.382	43	70, 61, 45	8.35 × 10^4^	1.10 × 10^6^	5.10 × 10^5^	0.17377	0.71429	0.057143	0.35238
Pyridine	110-86-1	46.200	79	52, 51, 50	9.96 × 10^4^	1.25 × 10^5^	3.18 × 10^5^	0.17764	0.2619	0.85714	0.11429
1-Propanol	71-23-8	26.439	59	42, 60, 41	0	1.26 × 10^6^	1.22 × 10^7^	0.20664	1	0.12274	0.1491
Hexanal	66-25-1	55.700	56	72, 82, 44	1.73 × 10^5^	1.73 × 10^5^	1.03 × 10^5^	0.22684	0.2619	0.85714	0.17143
* 3-Penten-2-one	*3102-33-8*	*48.521*	*69*	*84, 55, 41*	*3.34 × 10^3^*	*1.77 × 10^4^*	*4.96 × 10^4^*	*0.22984*	*0.89553*	*0.24189*	*0.13933*
Acetaldehyde	75-07-0	9.644	43	44, 42, 41	1.20 × 10^6^	1.42 × 10^6^	1.05 × 10^6^	0.23509	0.16667	0.4	0.47619
* Phenol	*108-95-2*	*67.000*	*94*	*66, 65, 39*	*5.05 × 10^5^*	*3.71 × 10^5^*	*5.85 × 10^5^*	*0.23638*	*0.16667*	*0.22857*	*0.91429*
n-Undecane	1120-21-4	73.800	57	43, 71, 85	3.60 × 10^5^	5.11 × 10^5^	3.49 × 10^5^	0.23638	0.2619	0.11429	1
* 2-Heptene	*14686-13-6*	*49.945*	*69*	*98, 56, 41*	*3.29 × 10^4^*	*4.55 × 10^4^*	*5.41 × 10^4^*	*0.25555*	*0.71429*	*0.85714*	*0.087118*
* Methanethiol	*74-93-1*	*10.900*	*47*	*48, 45, 46*	*3.04 × 10^5^*	*2.60 × 10^5^*	*2.18 × 10^5^*	*0.27494*	*0.38095*	*0.85714*	*0.17143*
Methyl Methacrylate	4655-34-9	45.175	69	100, 41, 39	1.31 × 10^4^	6.75 × 10^3^	1.79 × 10^4^	0.28948	0.89685	0.057143	0.45417
* Propene	*115-07-1*	*7.250*	*41*	*39, 42, 44*	*2.77 × 10^5^*	*4.90 × 10^5^*	*3.92 × 10^5^*	*0.29287*	*0.54762*	*0.22857*	*0.35238*
Benzaldehyde	100-52-7	65.584	106	105, 77, 51	3.57 × 10^5^	3.48 × 10^5^	2.62 × 10^5^	0.31369	0.16667	0.4	0.91429
Acetone	67-64-1	18.266	58	57, 44, 117	8.32 × 10^6^	2.18 × 10^7^	8.78 × 10^6^	0.31369	0.2619	0.22857	1
Butyrolactone	96-48-0	58.200	86	42, 56, 41	1.39 × 10^4^	2.60 × 10^4^	2.38 × 10^4^	0.32869	0.54762	0.4	0.25714
2-Methylfuran	534-22-5	30.700	82	53, 81, 39	8.32 × 10^4^	5.96 × 10^4^	5.76 × 10^4^	0.35693	0.38095	0.85714	0.25714
2-Butenal	4170-30-3	35.863	70	41, 39, 69	2.80 × 10^4^	4.68 × 10^4^	3.29 × 10^4^	0.3717	0.38095	0.22857	0.83066
Butanal	123-72-8	31.105	57	72, 44, 39	3.47 × 10^4^	2.89 × 10^4^	3.45 × 10^4^	0.37399	0.54762	0.62857	0.25714
* 1-Methoxy-2-propanol	*107-98-2*	*43.910*	*45*	*75, 47, 57*	*1.11 × 10^6^*	*3.78 × 10^5^*	*0.00 × 10^0^*	*0.39161*	*0.23859*	*1*	*0.30743*
Pentanal	110-62-3	45.598	44	58, 41, 57	6.08 × 10^4^	7.82 × 10^4^	4.56 × 10^4^	0.40058	0.2619	0.85714	0.47619
n-Heptane	142-82-5	50.100	71	57, 43, 100	9.93 × 10^4^	1.75 × 10^5^	1.46 × 10^5^	0.42671	0.71429	0.4	0.35238
* 2-Propenenitrile	*107-13-1*	*21.062*	*53*	*52, 51, 50*	*2.53 × 10^4^*	*3.52 × 10^4^*	*3.11 × 10^4^*	*0.4533*	*0.90476*	*0.4*	*0.35238*
2-(n)-PentylFuran	3777-69-3	69.325	81	82, 53, 138	1.34 × 10^5^	1.34 × 10^5^	1.07 × 10^5^	0.45831	0.2619	0.85714	0.60952
* N,N-Dimethyl-formamide	*68-12-2*	*48.577*	*73*	*44, 58*	*1.80 × 10^5^*	*6.15 × 10^5^*	*1.13 × 10^6^*	*0.46937*	*0.69364*	*0.47553*	*0.33142*
Furan	110-00-9	17.916	68	39, 38, 40	3.82 × 10^4^	3.65 × 10^4^	2.88 × 10^4^	0.51719	0.38095	1	0.47619
* iso-Butane	*75-28-5*	*12.000*	*43*	*41*, *42, 39*	*1.78 × 10^5^*	*4.96 × 10^4^*	*3.92 × 10^4^*	*0.55244*	*0.54762*	*0.4*	*0.60952*
p-Xylene	106-42-3	60.939	91	106, 105, 77	3.51 × 10^5^	8.26 × 10^5^	4.19 × 10^5^	0.57727	0.54762	0.4	0.91429
2-Methylpropanal	78-84-2	28.588	72	41, 43, 39	8.41 × 10^5^	1.12 × 10^5^	6.02 × 10^4^	0.58204	0.38095	0.62857	0.91429
3-Methyl-1-Butanol	123-51-3	50.395	55	70, 42, 41	7.55 × 10^6^	0	1.18 × 10^6^	0.58778	0.37676	0.5637	1
Isoprene	78-79-5	24.927	67	68, 53, 39	1.09 × 10^5^	1.96 × 10^5^	1.11 × 10^5^	0.59498	0.38095	0.62857	0.91429
* 1,3-Butadiene	*106-99-0*	*12.987*	*54*	*39, 53, 50*	*3.43 × 10^4^*	*4.19 × 10^4^*	*3.23 × 10^4^*	*0.59661*	*0.54762*	*0.4*	*0.91429*
* 2-Methyl-1,3-dioxolane	*497-26-7*	*35.455*	*73*	*58, 45, 87*	*3.29 × 10^4^*	*3.33 × 10^4^*	*3.93 × 10^4^*	*0.60653*	*0.54762*	*1*	*0.47619*
* 1-Butene	*106-98-9*	*13.659*	*56*	*55, 53, 50*	*2.33 × 10^4^*	*1.63 × 10^4^*	*1.37 × 10^4^*	*0.61831*	*0.38095*	*0.85714*	*0.7619*
1-Heptene	592-76-7	49.100	69	98, 70, 56	1.10 × 10^5^	8.79 × 10^4^	1.18 × 10^5^	0.61831	0.38095	0.85714	0.7619
* Trichloromethane	*67-66-3*	*31.000*	*83*	*85, 47*	*4.80 × 10^5^*	*9.13 × 10^4^*	*5.50 × 10^4^*	*0.64079*	*1*	*0.85714*	*0.35238*
Propanal	123-38-6	18.263	57	55, 39, 37	1.19 × 10^5^	2.72 × 10^5^	1.95 × 10^5^	0.66227	0.38095	0.85714	0.91429
Benzene	71-43-2	38.137	78	77, 51, 52	4.85 × 10^5^	5.44 × 10^5^	5.08 × 10^5^	0.66227	0.71429	0.4	0.91429
(E)-2-Methyl-2-Butenal	1115-11-3	47.964	84	55, 39, 41	6.55 × 10^4^	2.63 × 10^4^	9.62 × 10^3^	0.67383	0.51148	1	0.58949
(E)-1,3-Pentadiene	2004-70-8	27.082	67	68, 53, 39	2.63 × 10^4^	3.11 × 10^4^	2.48 × 10^4^	0.69013	0.54762	1	0.60952
* (E)-2-Butene	*624-64-6*	*13.659*	*56*	*55, 53, 50*	*2.88 × 10^4^*	*2.19 × 10^4^*	*1.85 × 10^4^*	*0.69013*	*0.54762*	*0.62857*	*0.91429*
* n-Butane	*106-97-8*	*13.378*	*58*	*39, 57*	*1.32 × 10^4^*	*1.14 × 10^4^*	*1.08 × 10^4^*	*0.73716*	*0.90476*	*0.62857*	*0.60952*
* N-Methylpyrrole	*96-54-8*	*45.960*	*81*	*80, 39, 53*	*2.76 × 10^4^*	*2.54 × 10^4^*	*2.29 × 10^4^*	*0.76397*	*0.71429*	*0.85714*	*0.60952*
1-Hexene	592-41-6	36.000	41	84, 69, 56	1.31 × 10^5^	1.42 × 10^5^	1.34 × 10^5^	0.77453	0.71429	0.62857	0.91429
* 1,2-Propanediol	*57-55-6*	*47.175*	*45*	*43, 61, 57*	*1.30 × 10^7^*	*1.48 × 10^8^*	*4.54 × 10^7^*	*0.78076*	*0.59588*	*0.8255*	*1*
Ethylbenzene	100-41-4	60.000	91	106, 65, 77	3.90 × 10^5^	4.37 × 10^5^	3.98 × 10^5^	0.79174	0.71429	0.62857	0.91429
2-Pentanon	107-87-9	44.911	43	86, 71, 58	1.55 × 10^5^	4.72 × 10^5^	1.48 × 10^5^	0.79174	0.71429	0.62857	0.91429
(Z)-2-Pentene	627-20-3	25.271	55	70, 71, 56	2.35 × 10^4^	2.31 × 10^4^	3.96 × 10^4^	0.79174	0.90476	0.4	0.7619
Acetophenone	98-86-2	71.213	105	77, 120, 51	4.52 × 10^5^	5.19 × 10^5^	6.43 × 10^5^	0.80998	0.79542	1	0.60952
Ethanol	64-17-5	14.000	45	46, 43	1.10 × 10^7^	5.69 × 10^5^	3.59 × 10^7^	0.82053	1	0.62857	0.7619
Acetonitrile	75-05-8	14.852	41	40, 39, 38	4.95 × 10^5^	5.01 × 10^5^	6.66 × 10^5^	0.82505	1	0.85714	0.60952
Methacrolein	78-85-3	28.261	70	41, 39, 42	6.86 × 10^4^	5.54 × 10^4^	5.92 × 10^4^	0.84107	1	0.4	1
2-Ethylacrolein	922-63-4	41.552	55	84, 56, 39	6.40 × 10^4^	2.98 × 10^4^	3.16 × 10^4^	0.90335	0.90476	0.85714	0.7619
n-Dodecane	112-40-3	76.800	57	71, 43, 85	2.34 × 10^5^	2.04 × 10^5^	2.04 × 10^5^	0.91082	0.90476	1	0.7619
n-Hexane	110-54-3	37.800	86	57, 43, 41	9.79 × 10^3^	1.33 × 10^4^	9.36 × 10^3^	0.91082	0.90476	1	0.7619
n-Pentane	109-66-0	25.545	72	57, 43, 41	1.17 × 10^4^	1.07 × 10^4^	9.37 × 10^3^	0.92517	1	0.62857	1
(E)-2-Pentene	646-04-8	25.680	55	70, 53, 56	1.02 × 10^5^	1.14 × 10^5^	1.13 × 10^5^	0.92596	0.90476	0.85714	0.91429
1-Dodecene	112-41-4	76.440	55	69, 83, 97	6.21 × 10^4^	4.61 × 10^4^	6.73 × 10^4^	0.93261	0.89274	0.85445	0.91406
Heptanal	111-71-7	63.800	81	70, 43, 55	2.40 × 10^4^	2.24 × 10^4^	2.20 × 10^4^	0.93619	0.71429	1	1
* 2-Methyl-1-propene	115-11-7	12.629	56	41, 39, 55	3.55 × 10^5^	2.94 × 10^5^	3.01 × 10^5^	0.93619	0.90476	0.85714	0.91429
Dimethyl Disulfide	624-92-0	47.136	94	43, 79, 61	7.54 × 10^3^	6.61 × 10^3^	6.14 × 10^3^	0.93816	0.89274	0.84536	1
4-Heptanone	123-19-3	62.200	71	43, 114, 41	3.16 × 10^4^	3.34 × 10^3^	3.79 × 10^4^	0.94038	0.87809	1	0.89565
2-Methylpyrazine	109-08-0	54.046	94	67, 53, 40	2.46 × 10^4^	1.83 × 10^4^	2.35 × 10^4^	0.94654	1	1	0.7619
3-Methyl-1-Butene	563-45-1	24.148	55	70, 41, 42	2.01 × 10^5^	2.14 × 10^5^	2.43 × 10^5^	0.94654	0.90476	0.85714	0.91429
* Isobutyronitrile	*78-82-0*	*34.940*	*68*	*42, 54, 52*	*4.14 × 10^4^*	*3.94 × 10^4^*	*4.23 × 10^4^*	*0.95175*	*1*	*0.85714*	*0.91429*
* (Z)-2-Butene	*590-18-1*	*12.766*	*41*	*56, 39, 55*	*2.01 × 10^5^*	*1.94 × 10^5^*	*1.68 × 10^5^*	*0.95175*	*0.90476*	*0.62857*	*1*
2-Heptanone	110-43-0	63.000	58	43, 71, 114	6.20 × 10^4^	5.34 × 10^4^	6.61 × 10^4^	0.95963	0.90476	1	0.91429
Toluene	108-88-3	51.942	91	92, 65, 39	1.18 × 10^6^	1.20 × 10^6^	1.06 × 10^6^	1	0.90476	0.85714	0.91429
D-Limoinene	5989-27-5	71.250	68	93, 67, 136	1.07 × 10^5^	5.58 × 10^5^	1.87 × 10^5^	1	1	1	1

## Data Availability

The dataset used and analyzed during the current study is available from the corresponding author upon reasonable request.

## Supplementary Materials

The following supporting information can be downloaded at: https://www.mdpi.com/article/10.3390/biom14070788/s1, Figure S1: C_albicans LOG; Figure S2: C_albicans STEADY; Figure S3: S_aureus LOG; Figure S4: S_aureus STEADY; Figure S5: CO-Culture LOG; Figure S6: CO-Culture STEADY.
