# Supplementary figures and images for: Molecular Analysis of Volatile Metabolites Synthesized by Candida albicans and Staphylococcus aureus in In Vitro Cultures and Bronchoalveolar Lavage Specimens Reflecting Single- or Duo-Factor Pneumonia
Source: Biomolecules. 2024 Jul 2;14(7):788. doi: 10.3390/biom14070788 (PMC11275233; doi:10.3390/biom14070788)

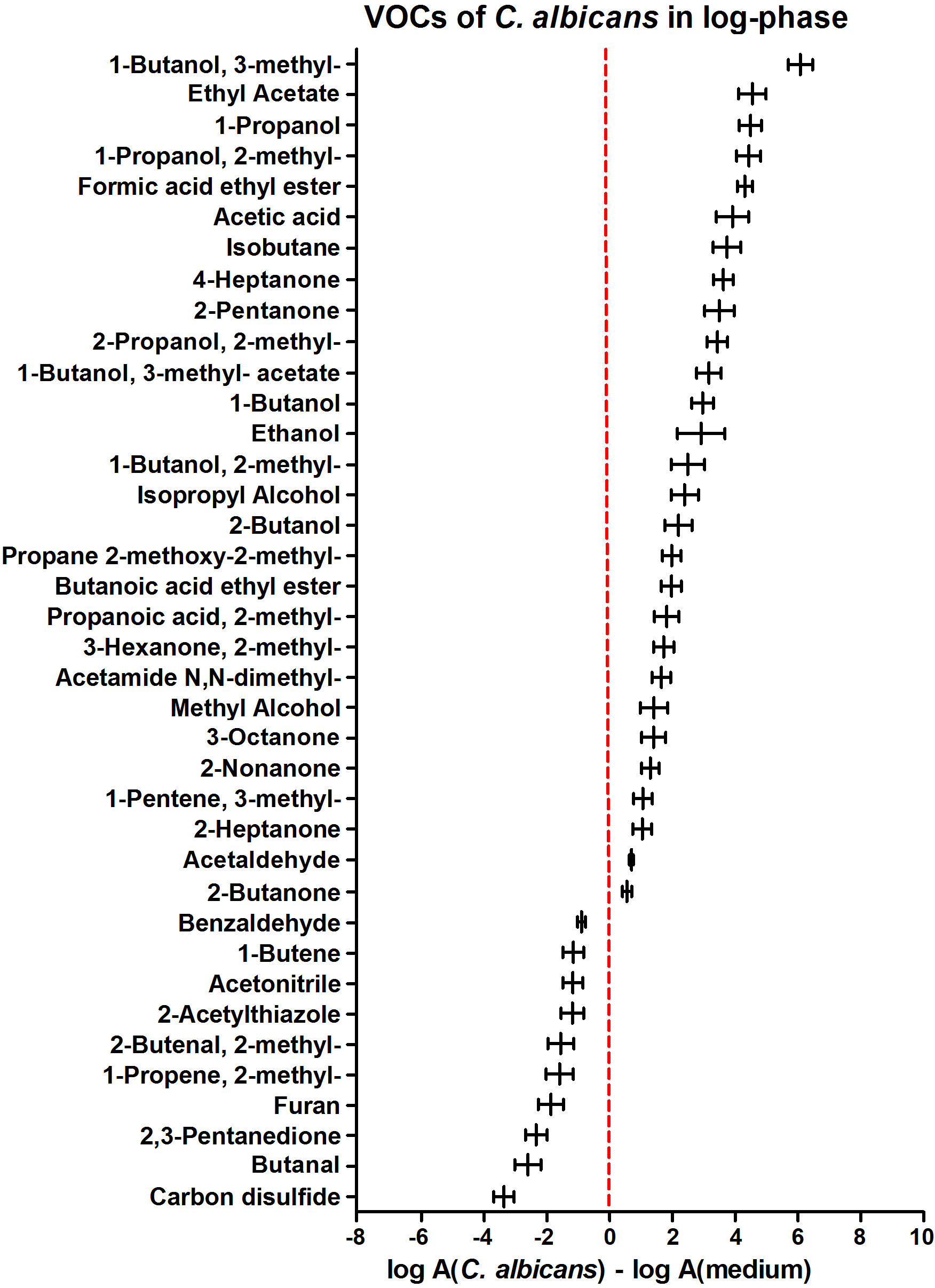

Supplement: Supplementary file 1 [file biomolecules-14-00788-s001.zip › Supplementary Figure 1 - C_albicans LOG-phase.png]

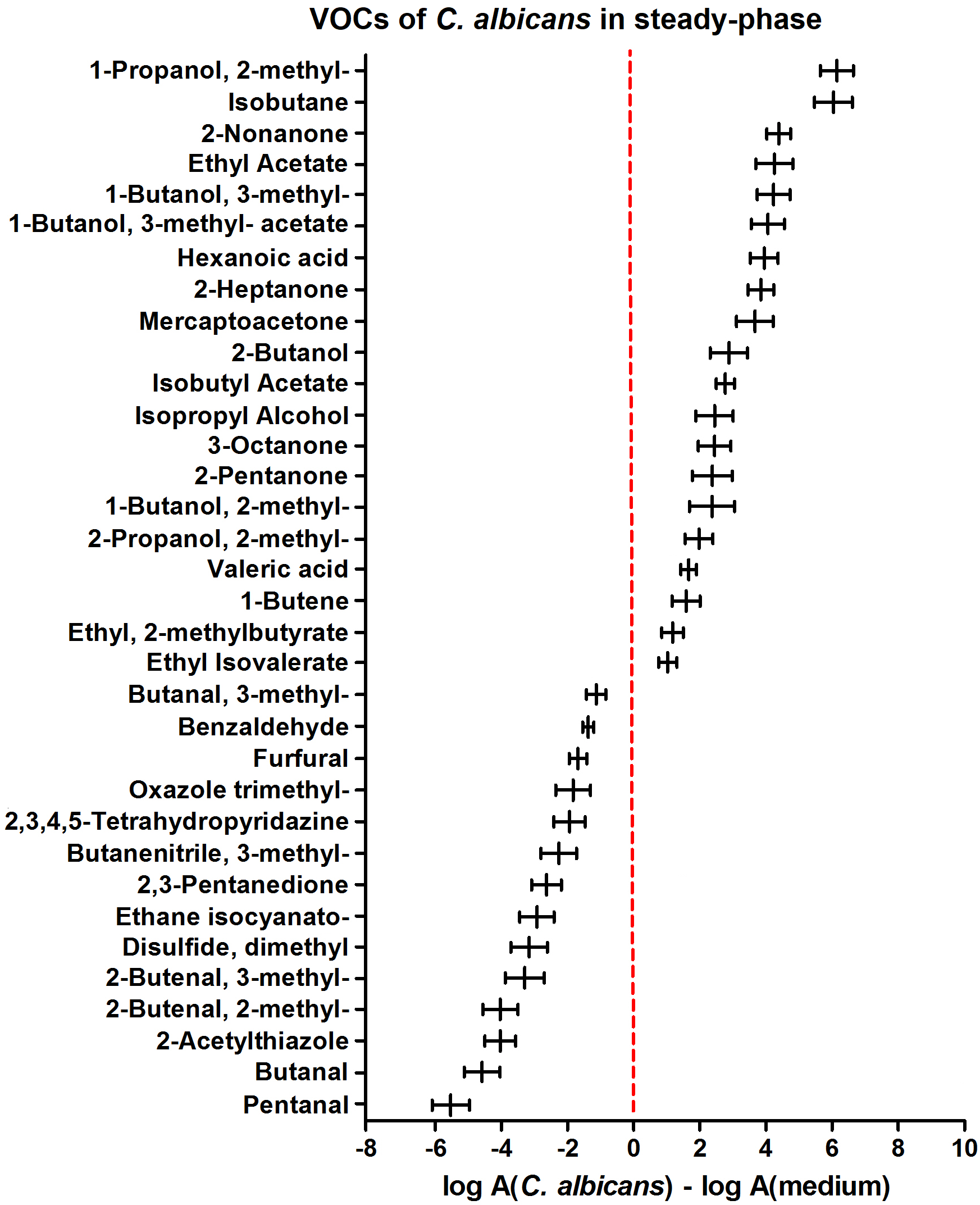

Supplement: Supplementary file 1 [file biomolecules-14-00788-s001.zip › Supplementary Figure 2 - C_albicans STEADY-phase.png]

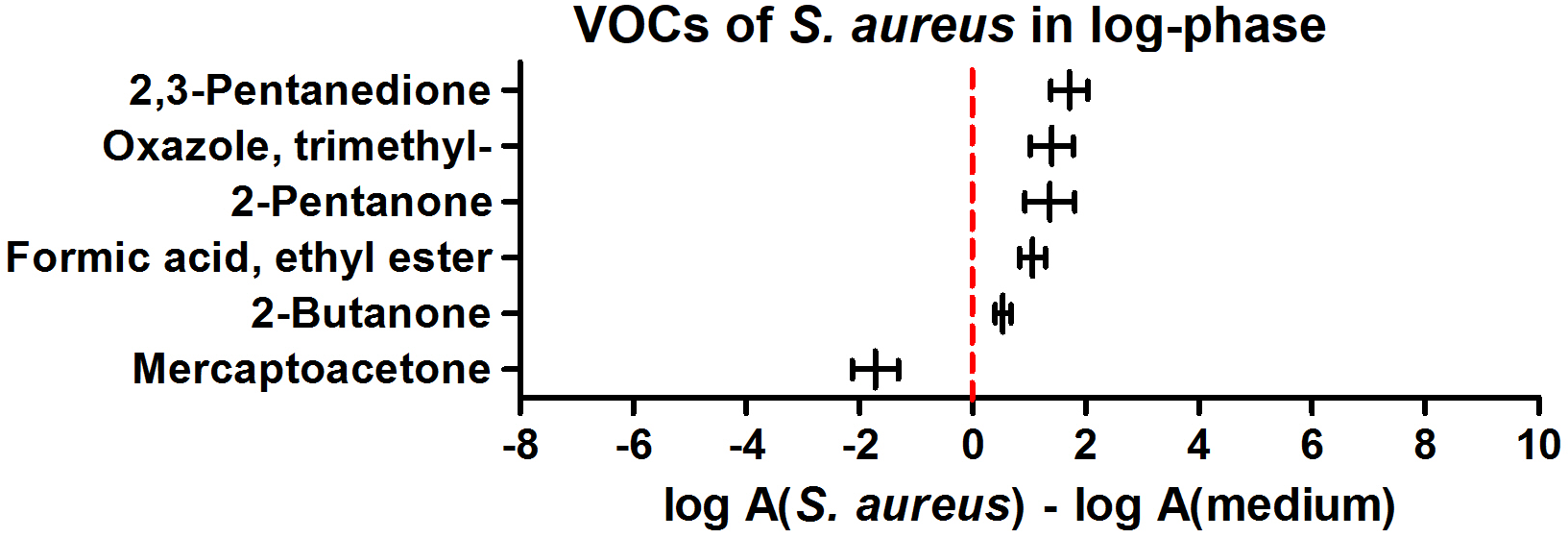

Supplement: Supplementary file 1 [file biomolecules-14-00788-s001.zip › Supplementary Figure 3 - S_aureus LOG-phase.png]

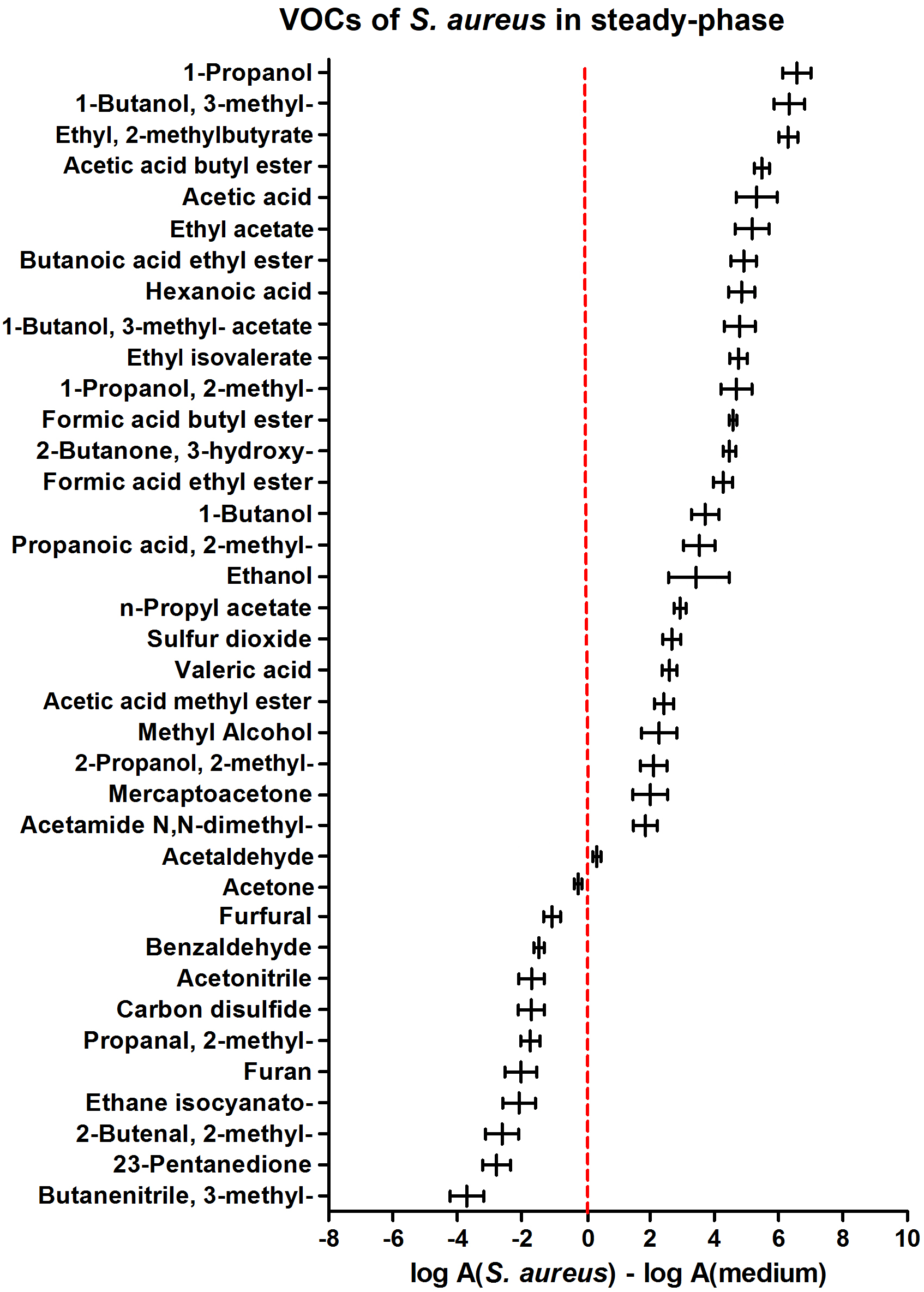

Supplement: Supplementary file 1 [file biomolecules-14-00788-s001.zip › Supplementary Figure 4 - S_aureus STEADY-phase.png]

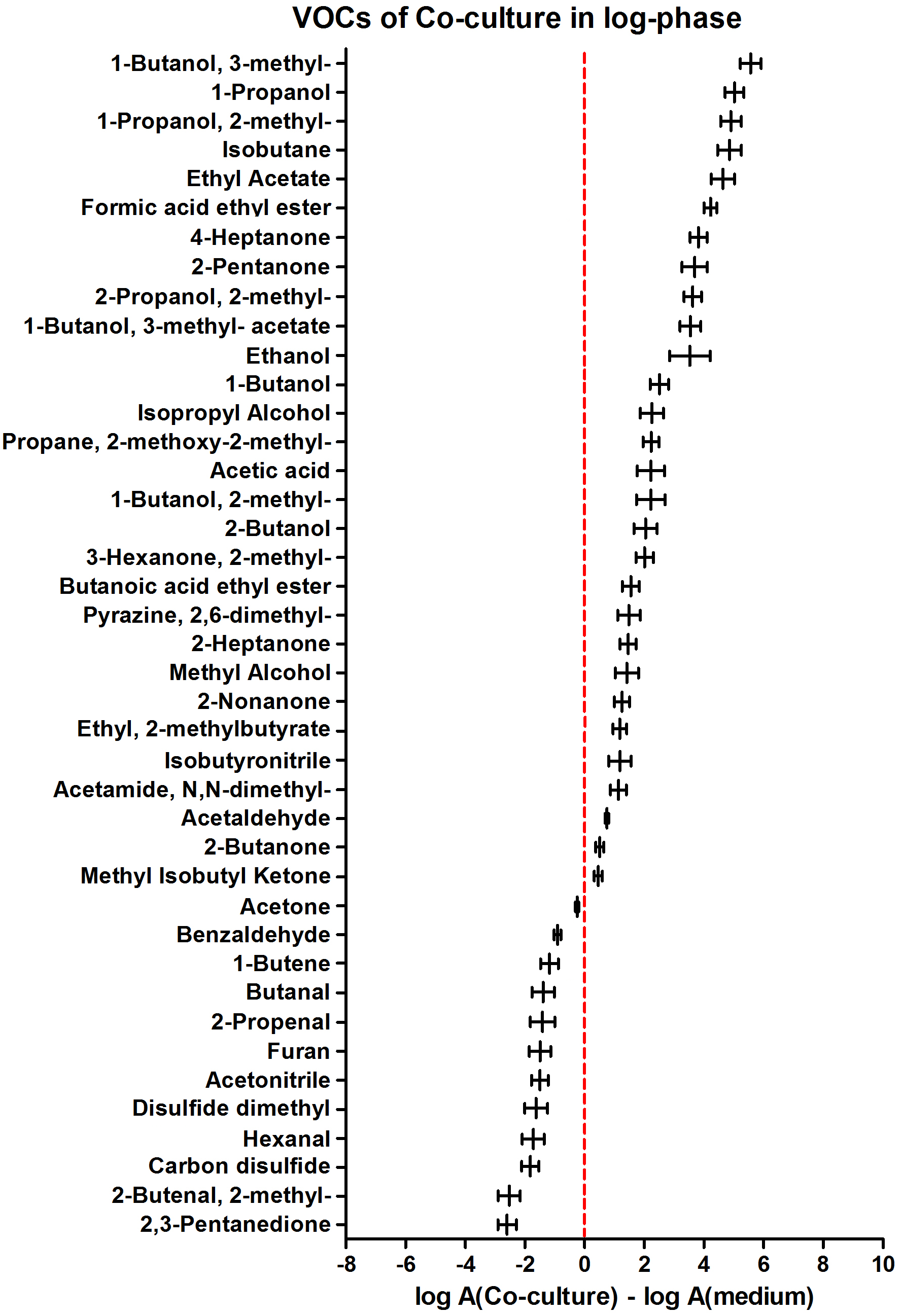

Supplement: Supplementary file 1 [file biomolecules-14-00788-s001.zip › Supplementary Figure 5 - CO-Culture LOG-phase.png]

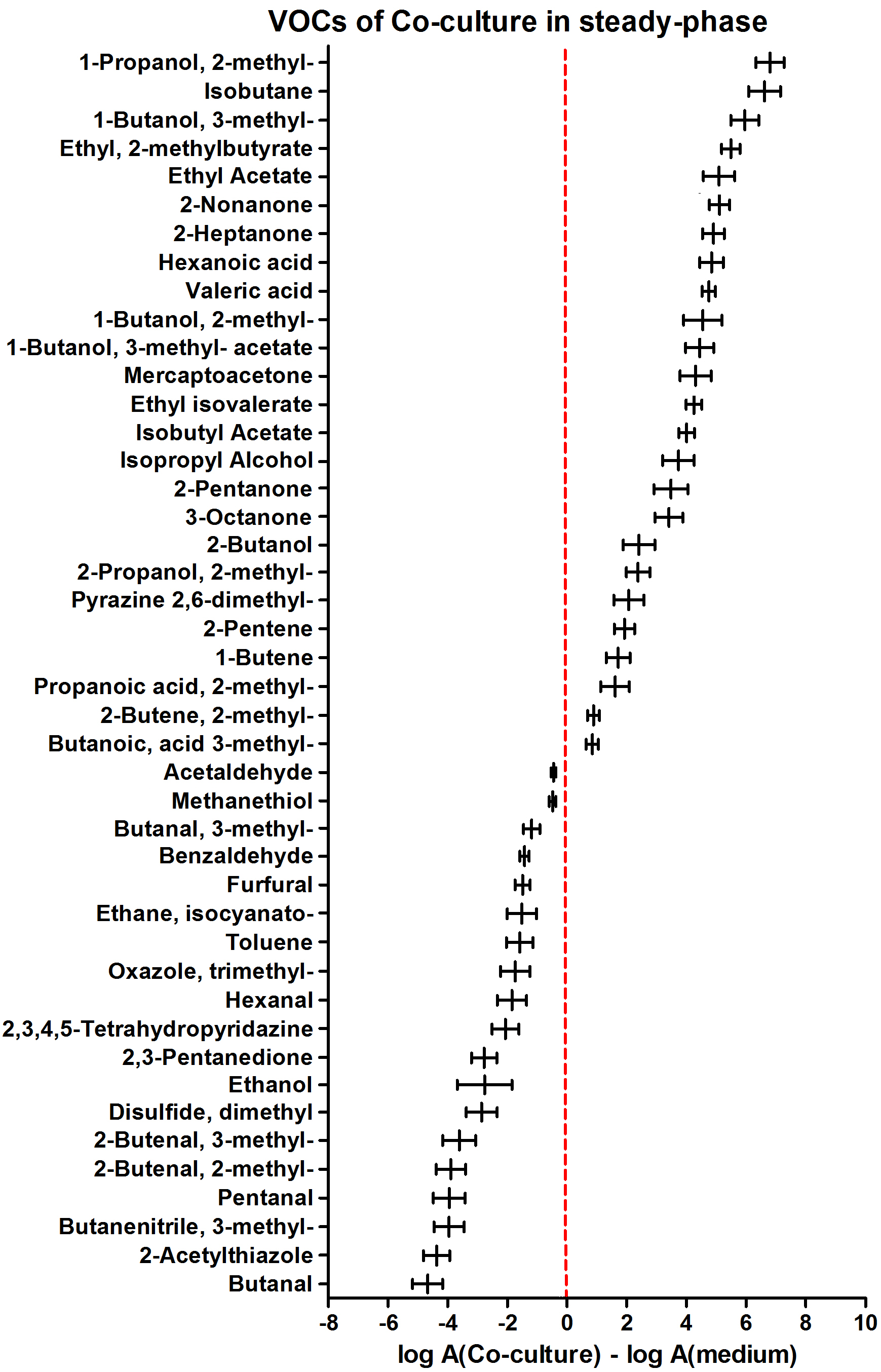

Supplement: Supplementary file 1 [file biomolecules-14-00788-s001.zip › Supplementary Figure 6 - CO-Culture STEADY-phase.png]
